# Supplementary material for: Anion channel SLAH3 is a regulatory target of chitin receptor-associated kinase PBL27 in microbial stomatal closure
Source: eLife. 2019 Sep 16;8:e44474. doi: 10.7554/eLife.44474 (PMC6776436; doi:10.7554/eLife.44474)
Supplement: Figure 2—source data 1. [file elife-44474-fig2-data1.pptx]

## Slide 1
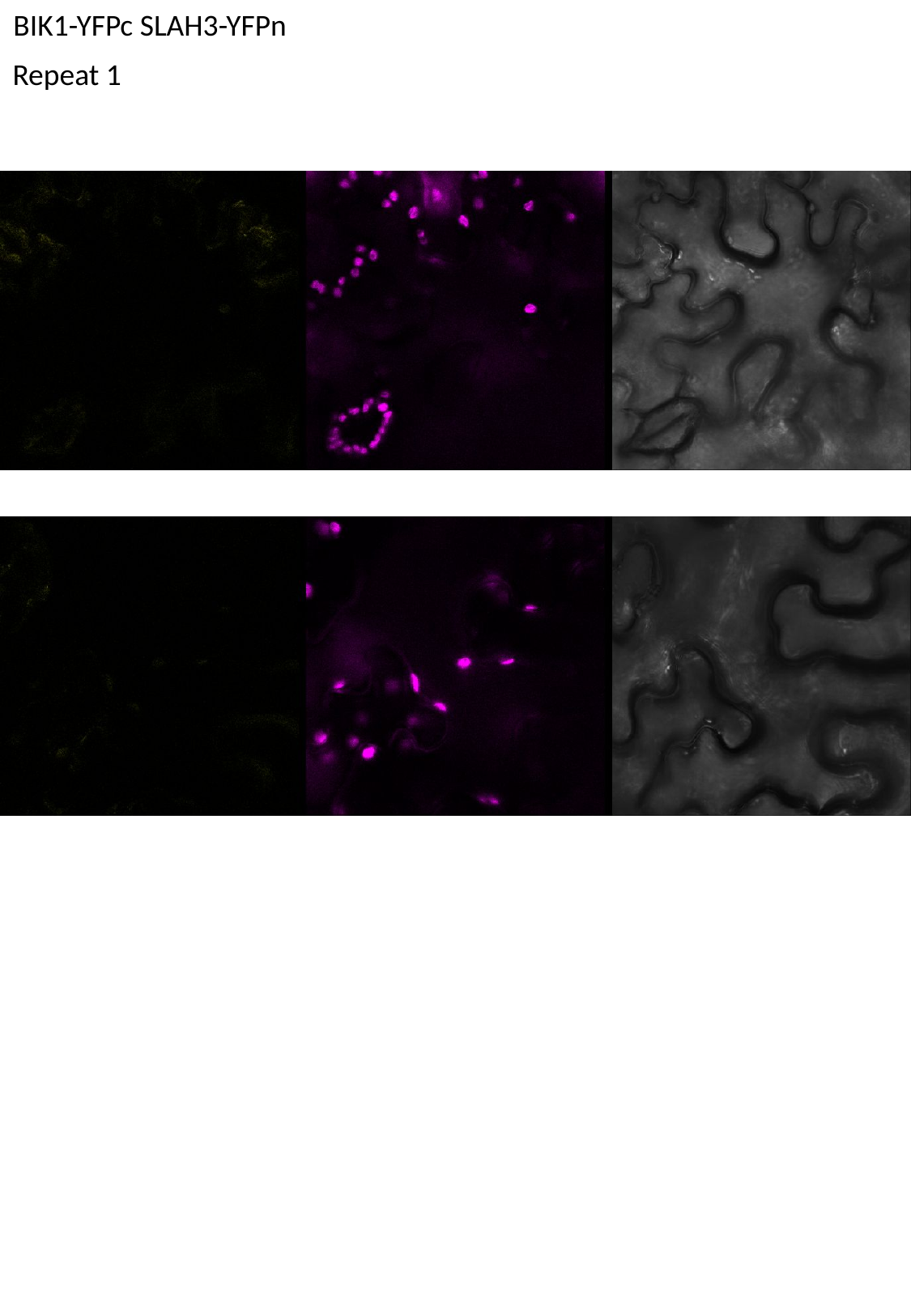

BIK1-YFPc SLAH3-YFPn
Repeat 1

## Slide 2
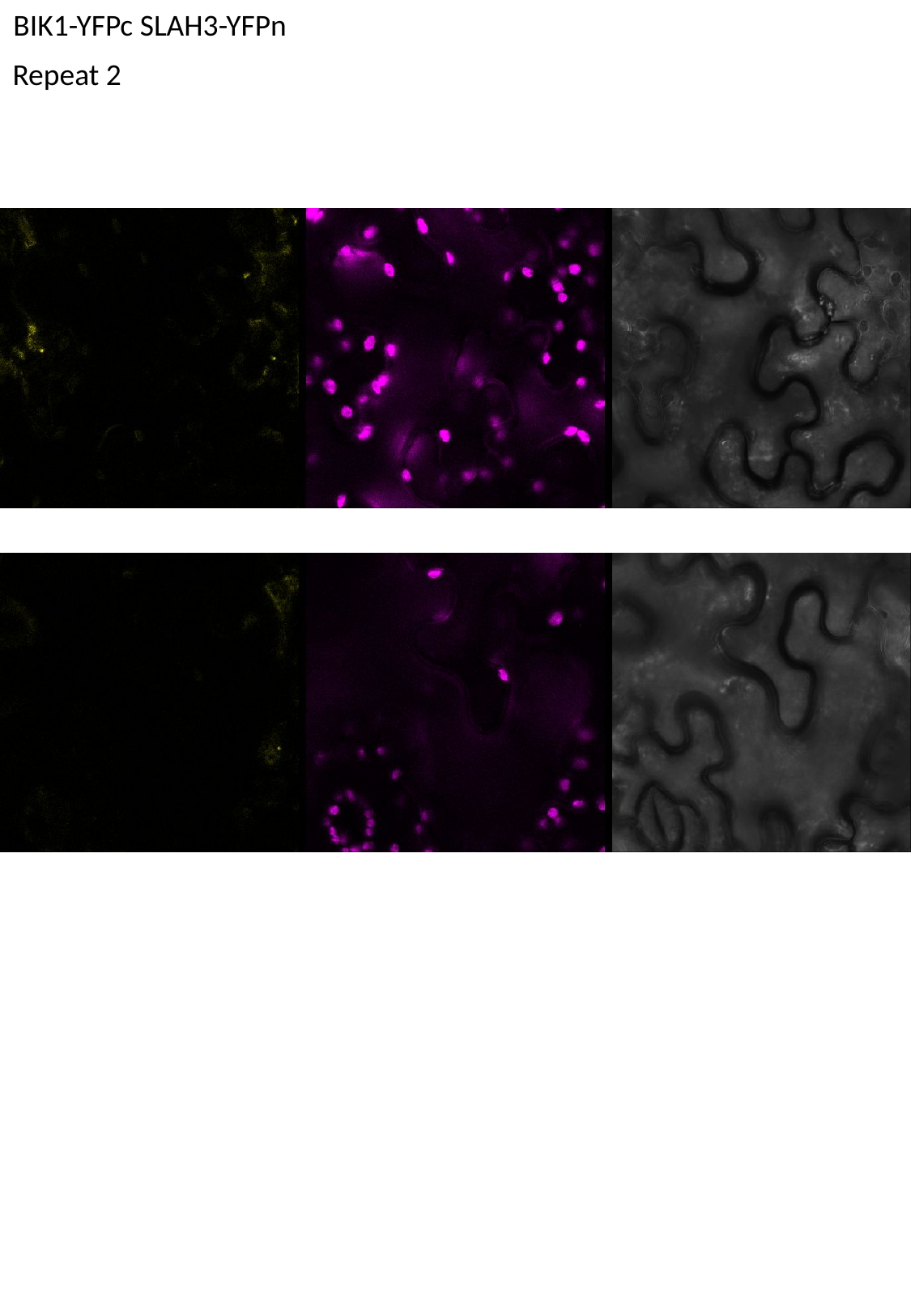

BIK1-YFPc SLAH3-YFPn
Repeat 2

## Slide 3
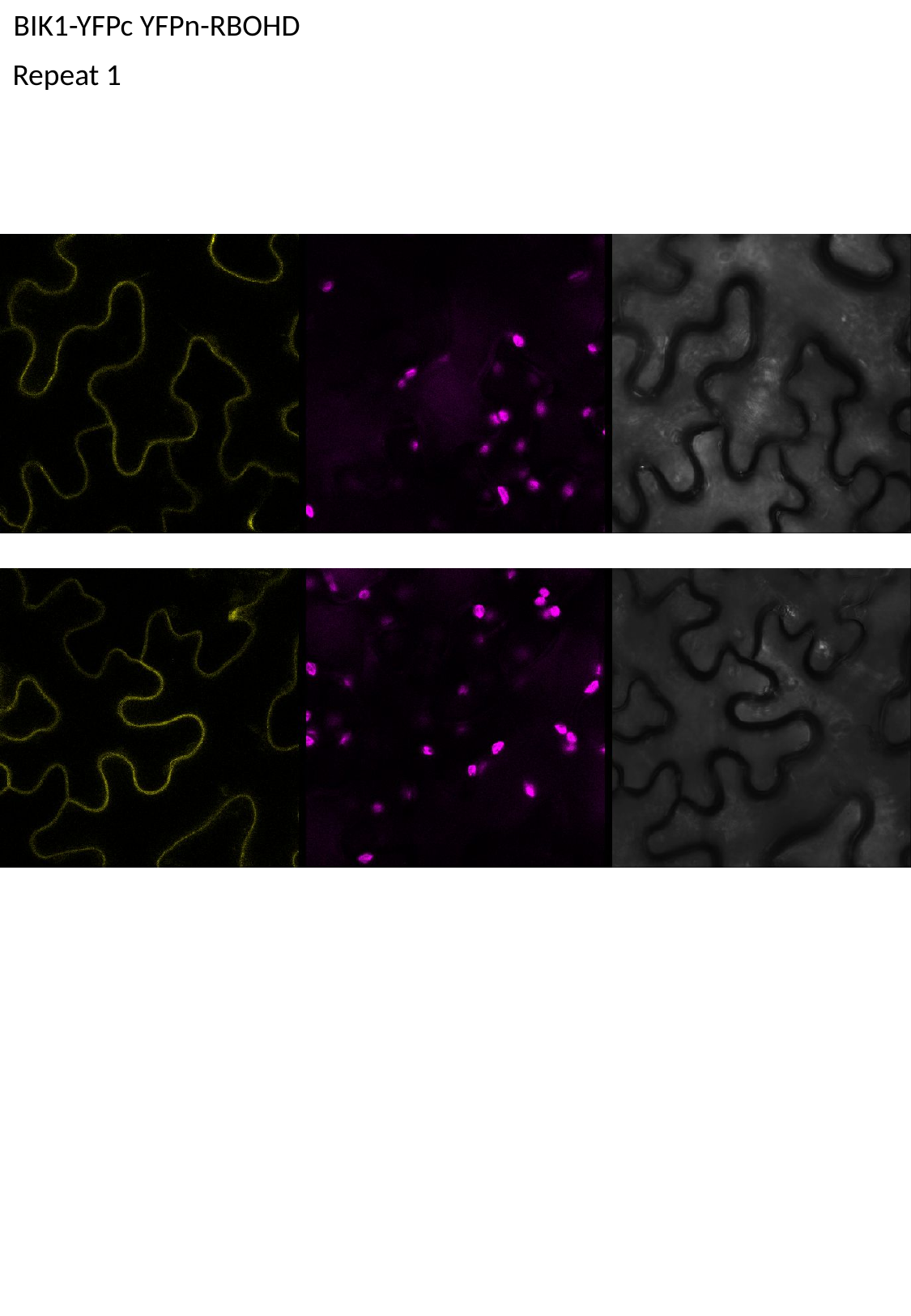

BIK1-YFPc YFPn-RBOHD
Repeat 1

## Slide 4
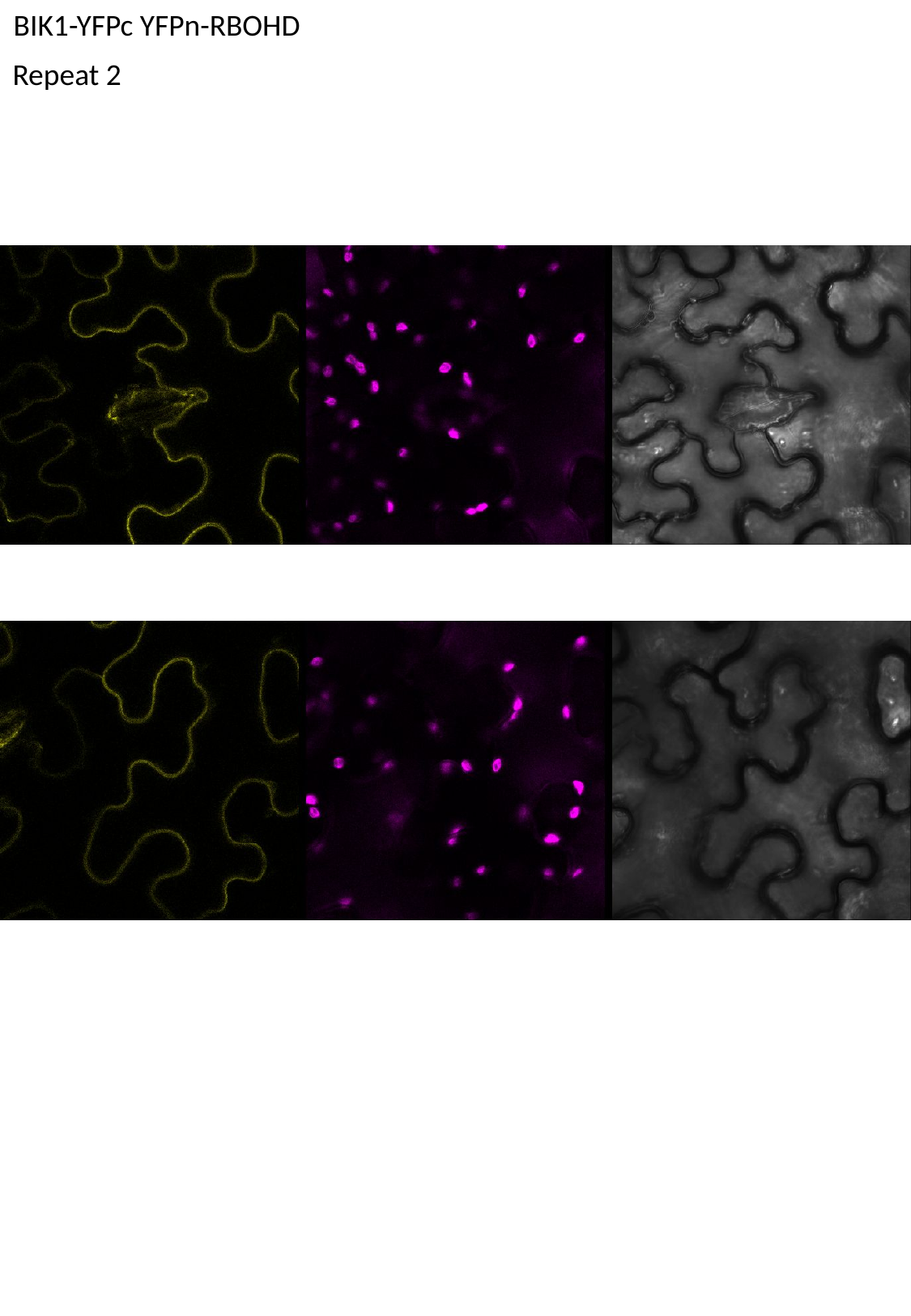

BIK1-YFPc YFPn-RBOHD
Repeat 2

## Slide 5
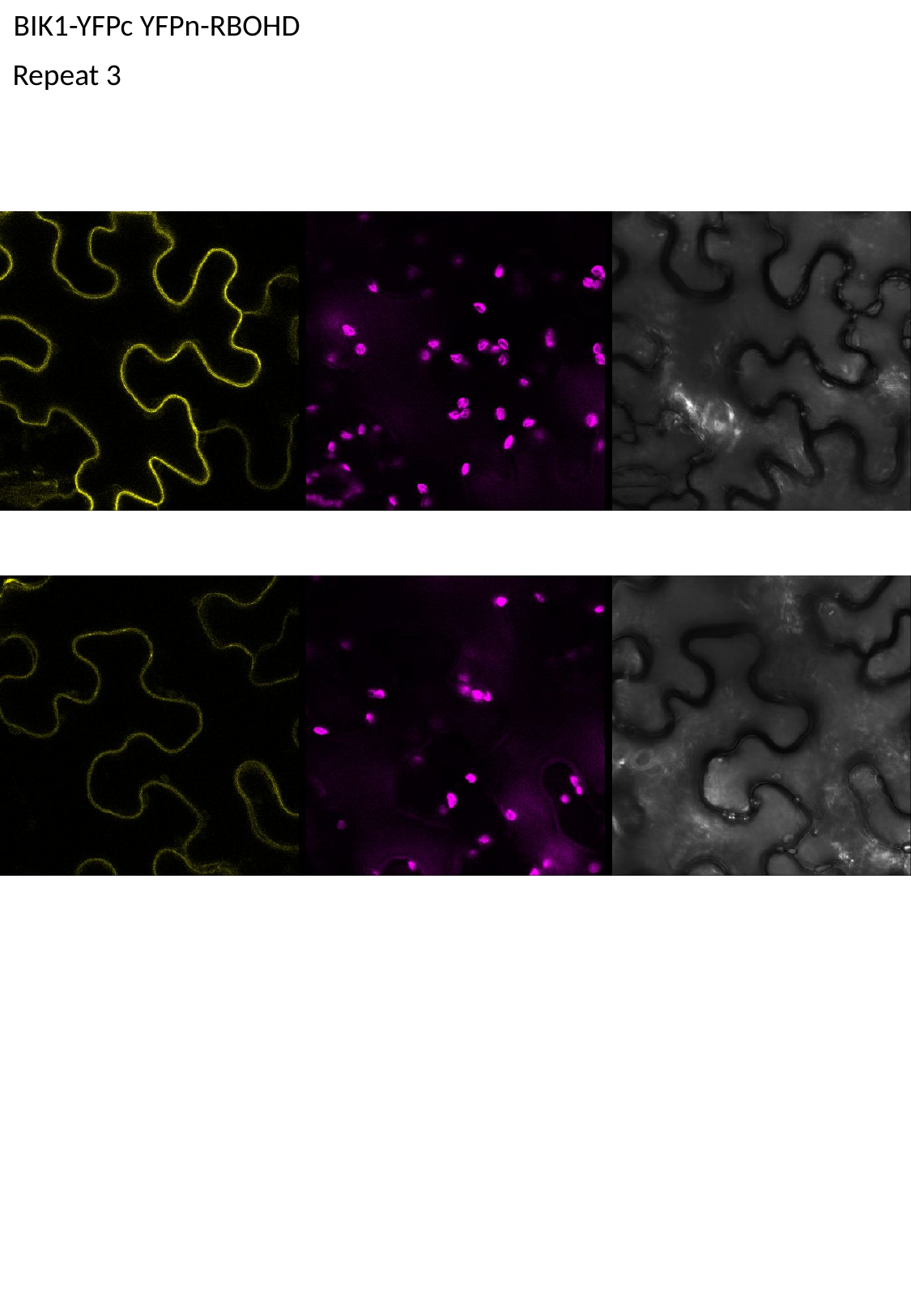

BIK1-YFPc YFPn-RBOHD
Repeat 3

## Slide 6
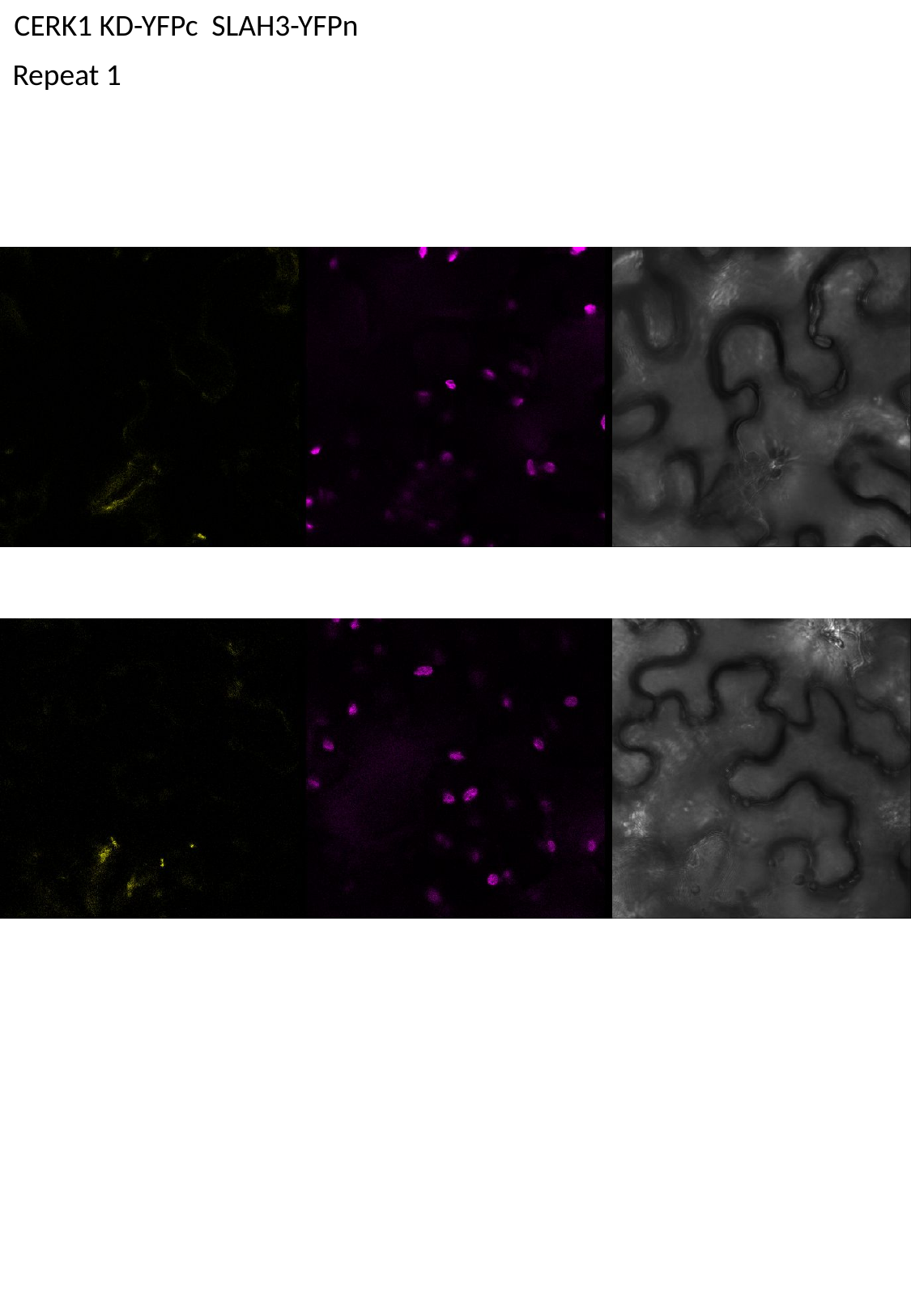

CERK1 KD-YFPc SLAH3-YFPn
Repeat 1

## Slide 7
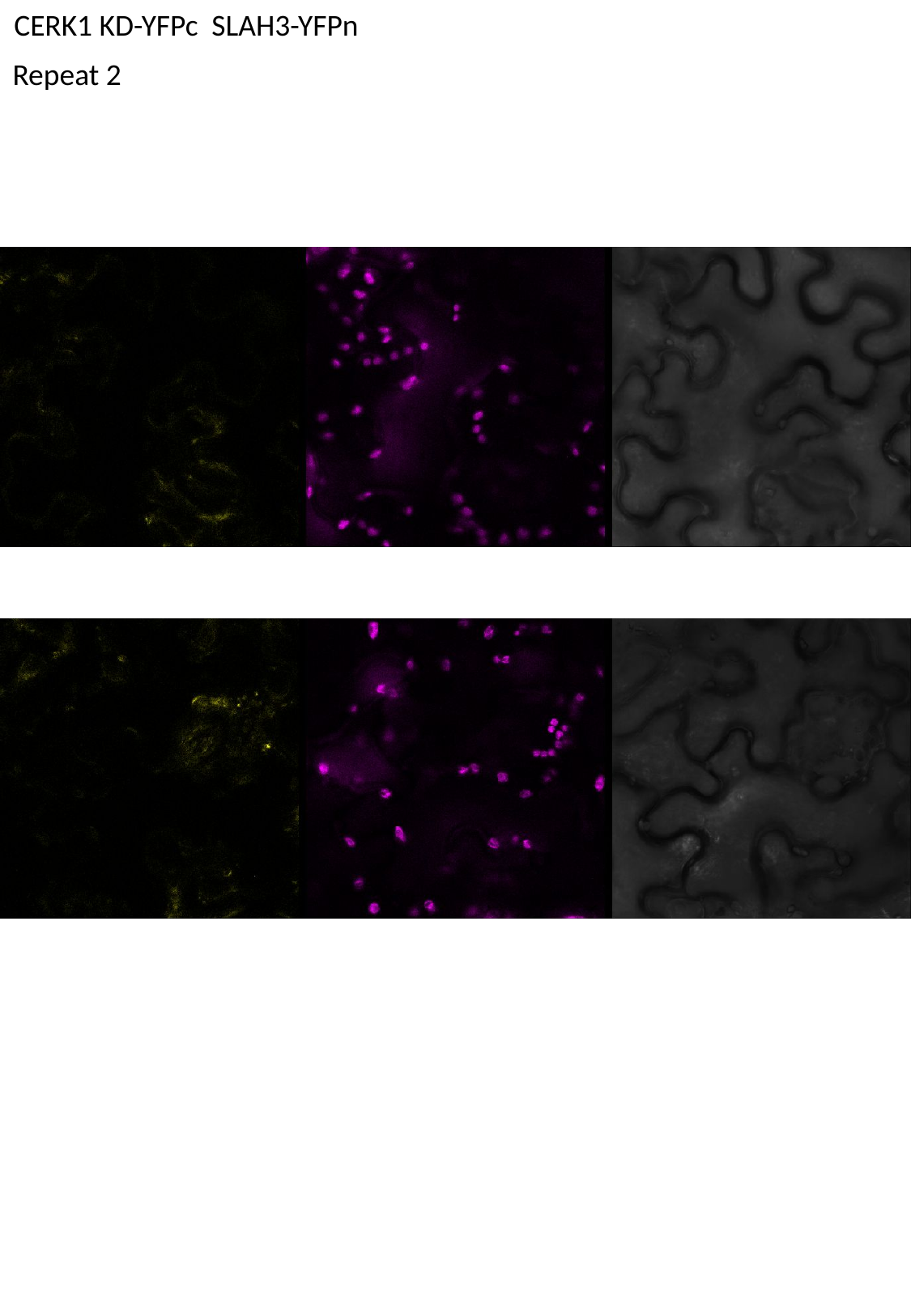

CERK1 KD-YFPc SLAH3-YFPn
Repeat 2

## Slide 8
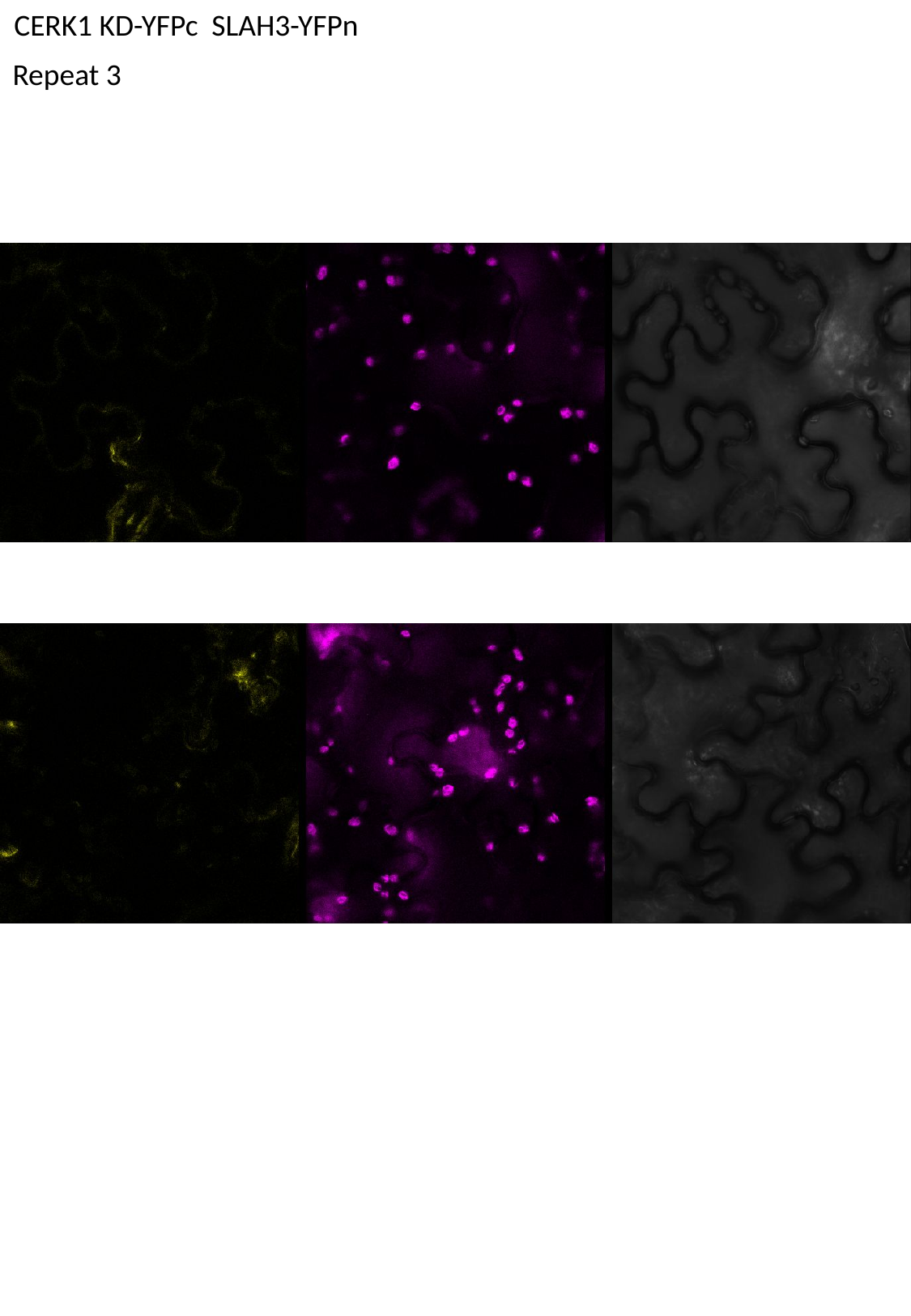

CERK1 KD-YFPc SLAH3-YFPn
Repeat 3

## Slide 9
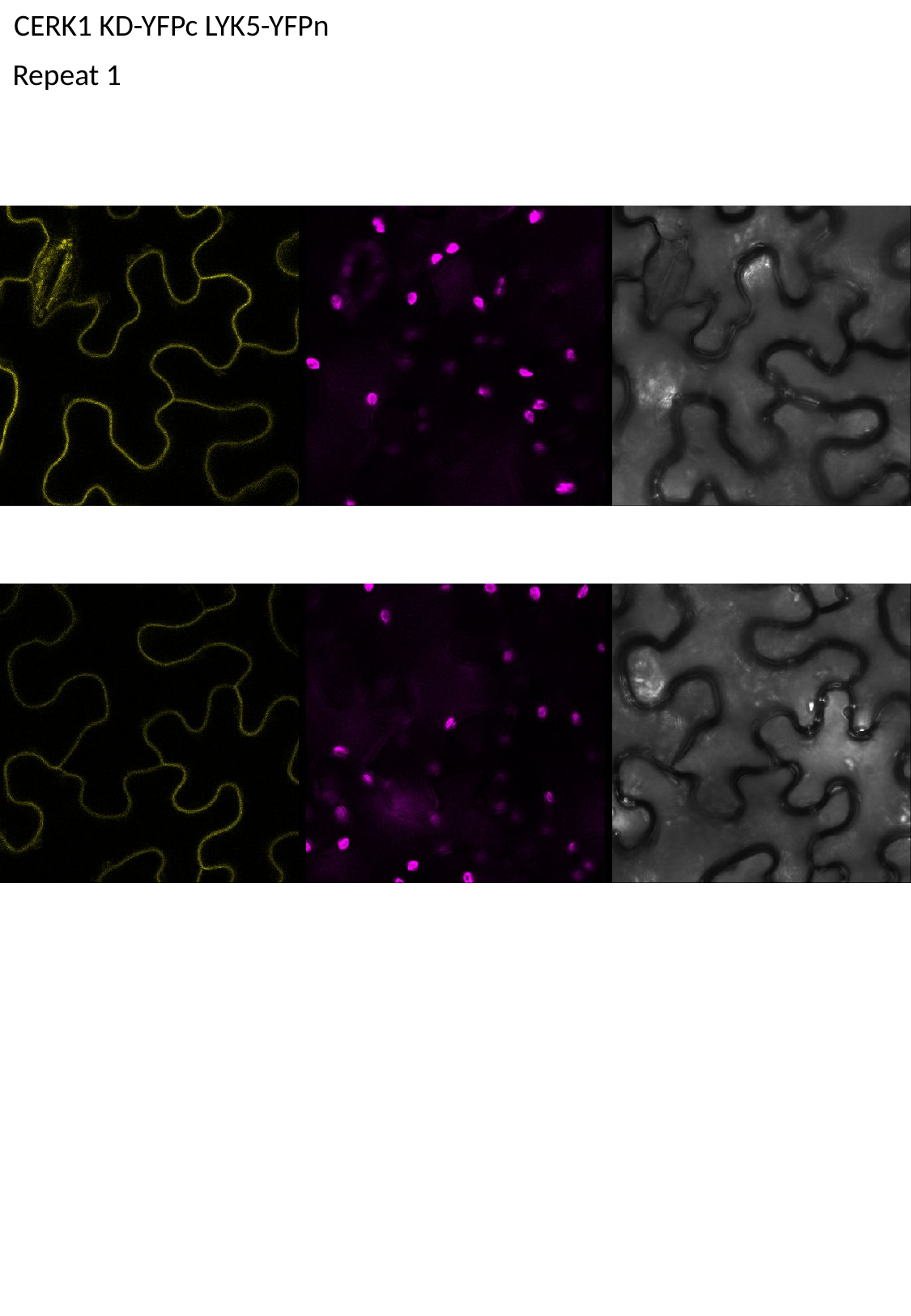

CERK1 KD-YFPc LYK5-YFPn
Repeat 1

## Slide 10
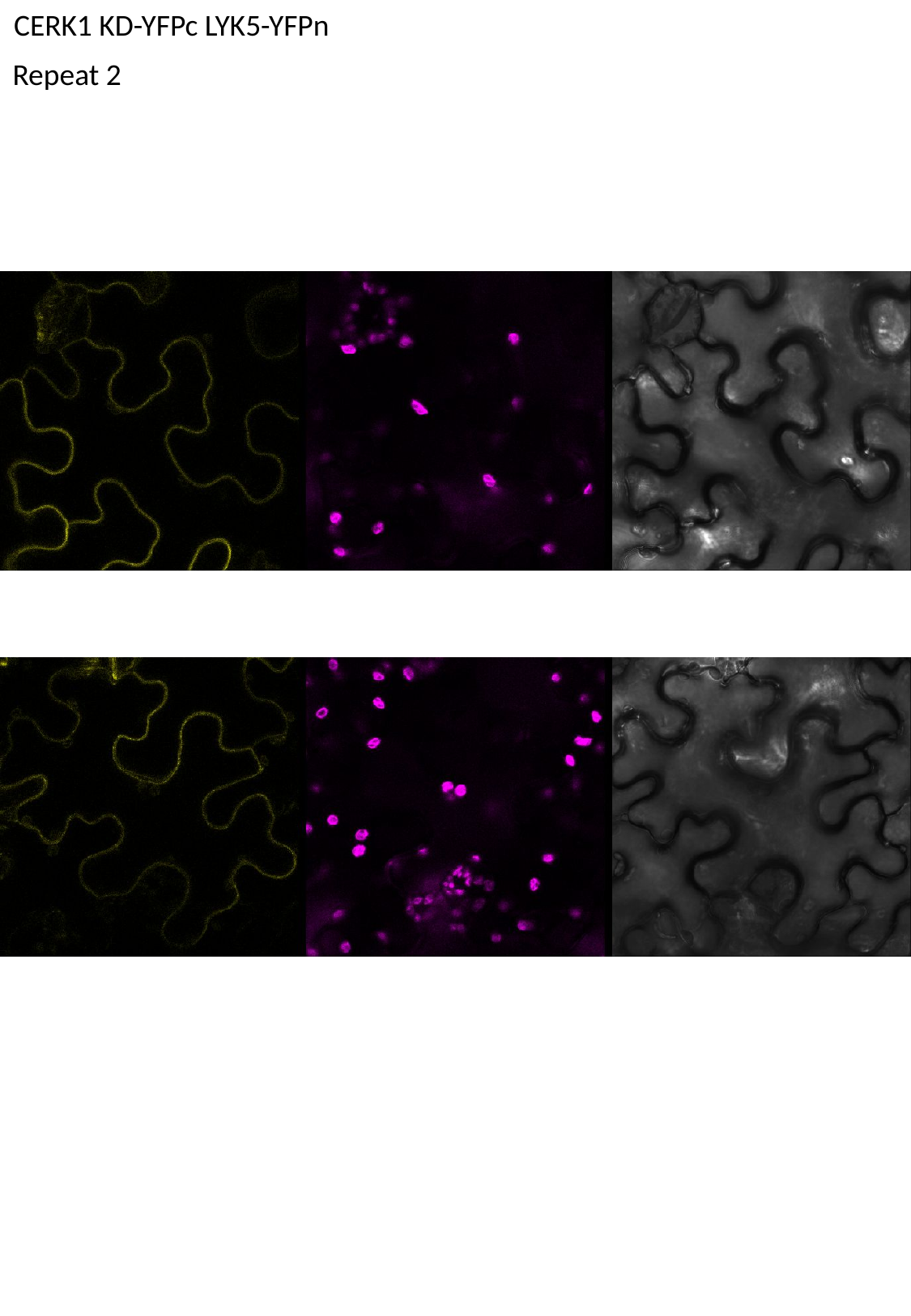

CERK1 KD-YFPc LYK5-YFPn
Repeat 2

## Slide 11
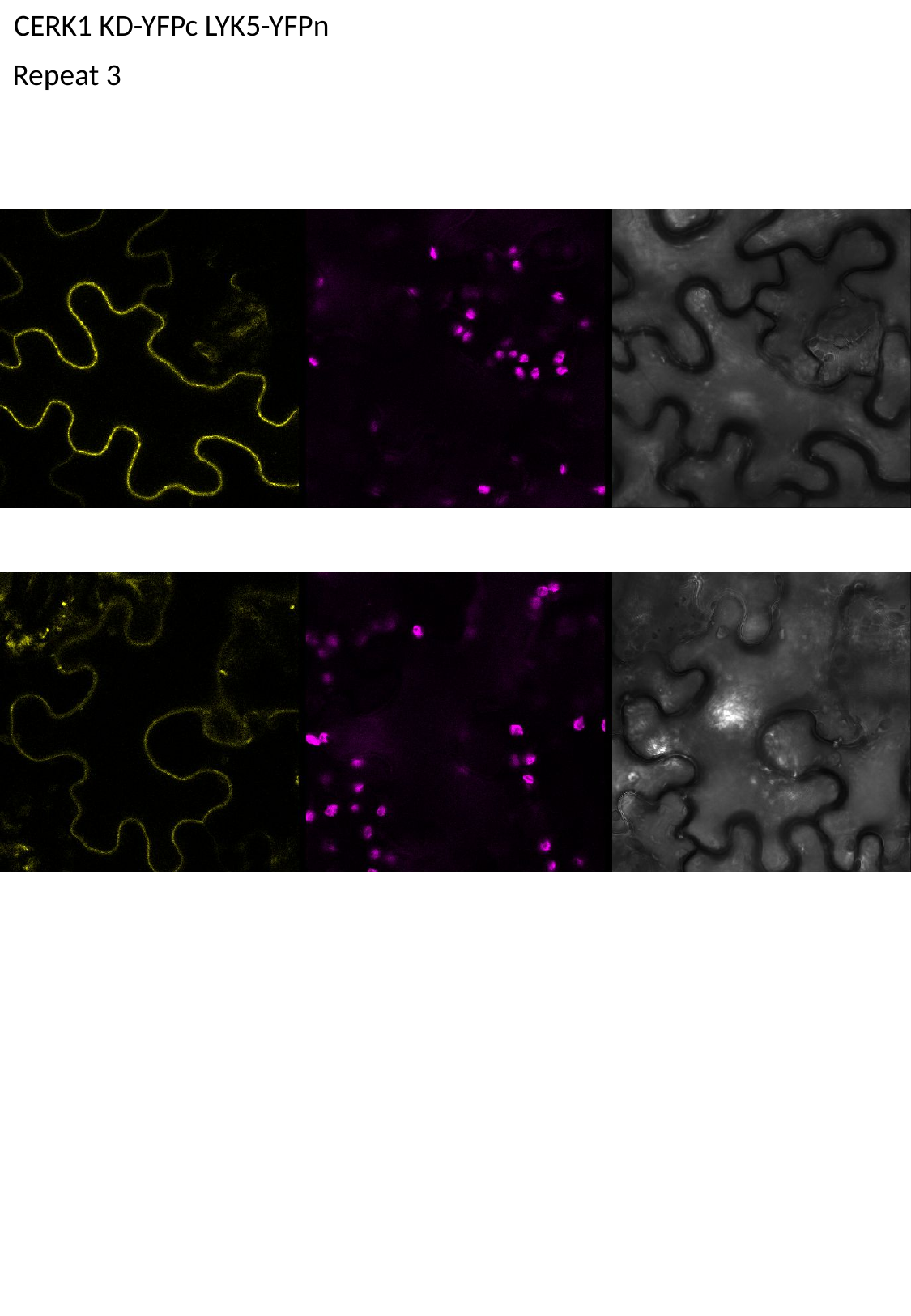

CERK1 KD-YFPc LYK5-YFPn
Repeat 3

## Slide 12
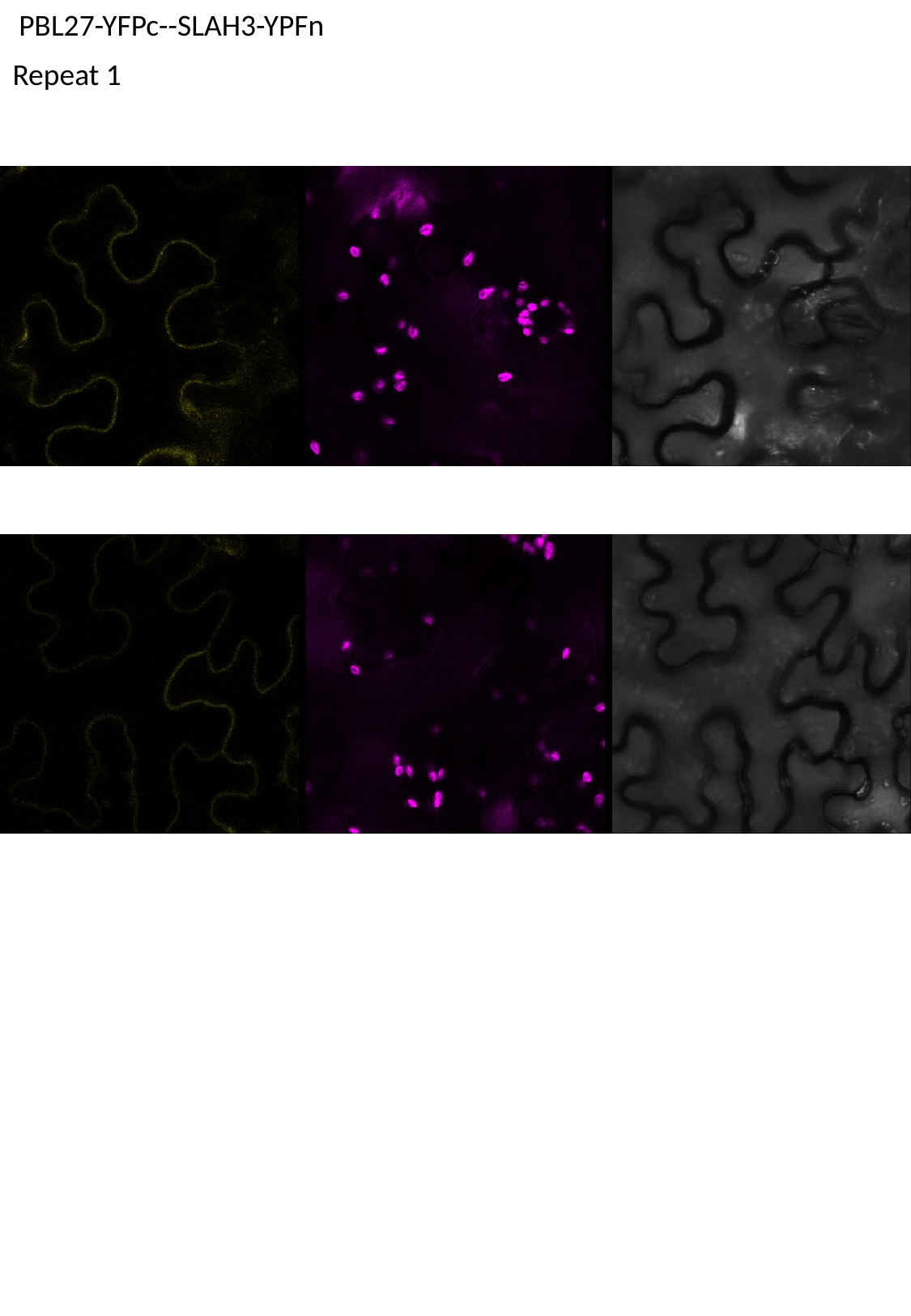

PBL27-YFPc--SLAH3-YPFn
Repeat 1

## Slide 13
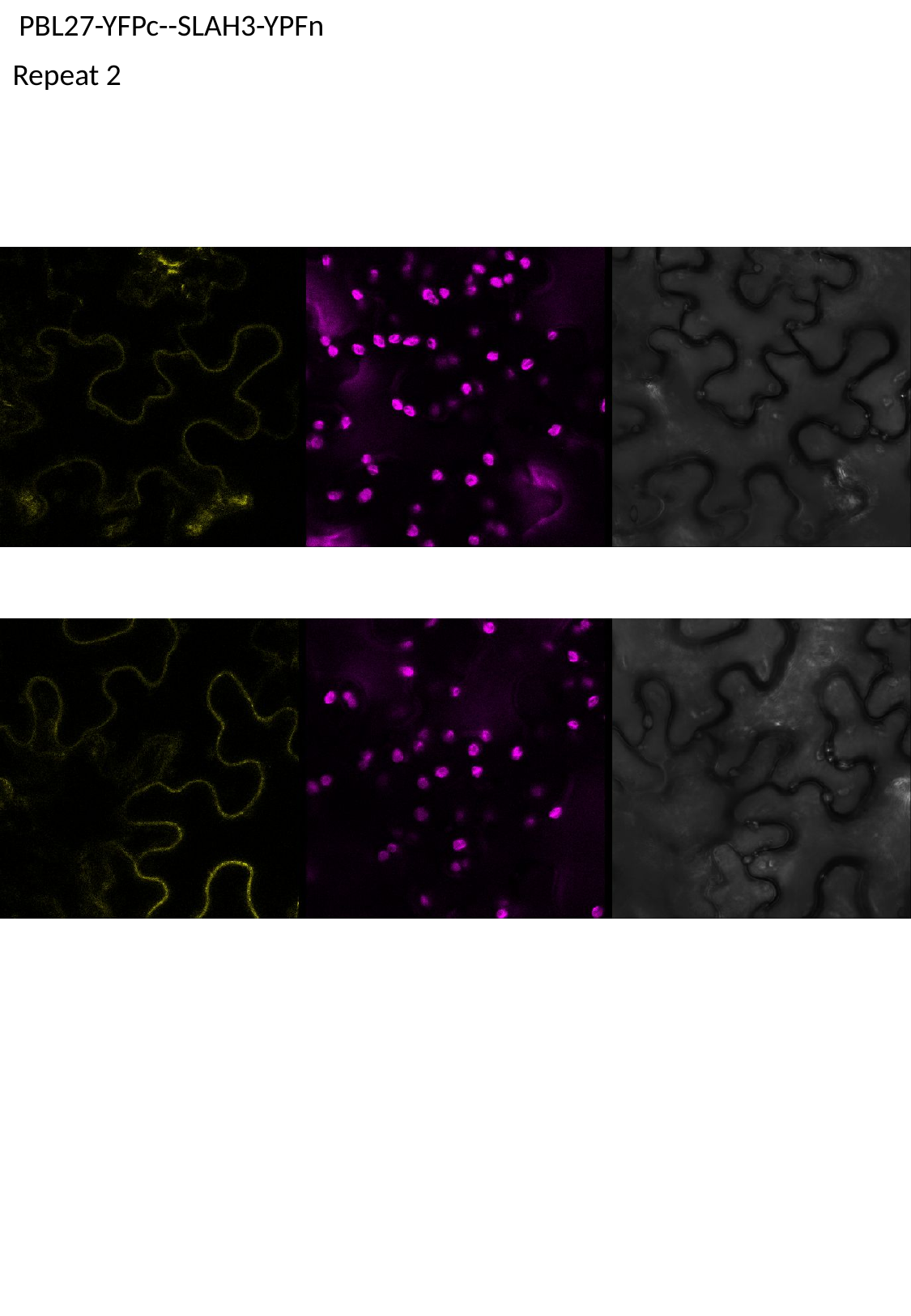

PBL27-YFPc--SLAH3-YPFn
Repeat 2

## Slide 14
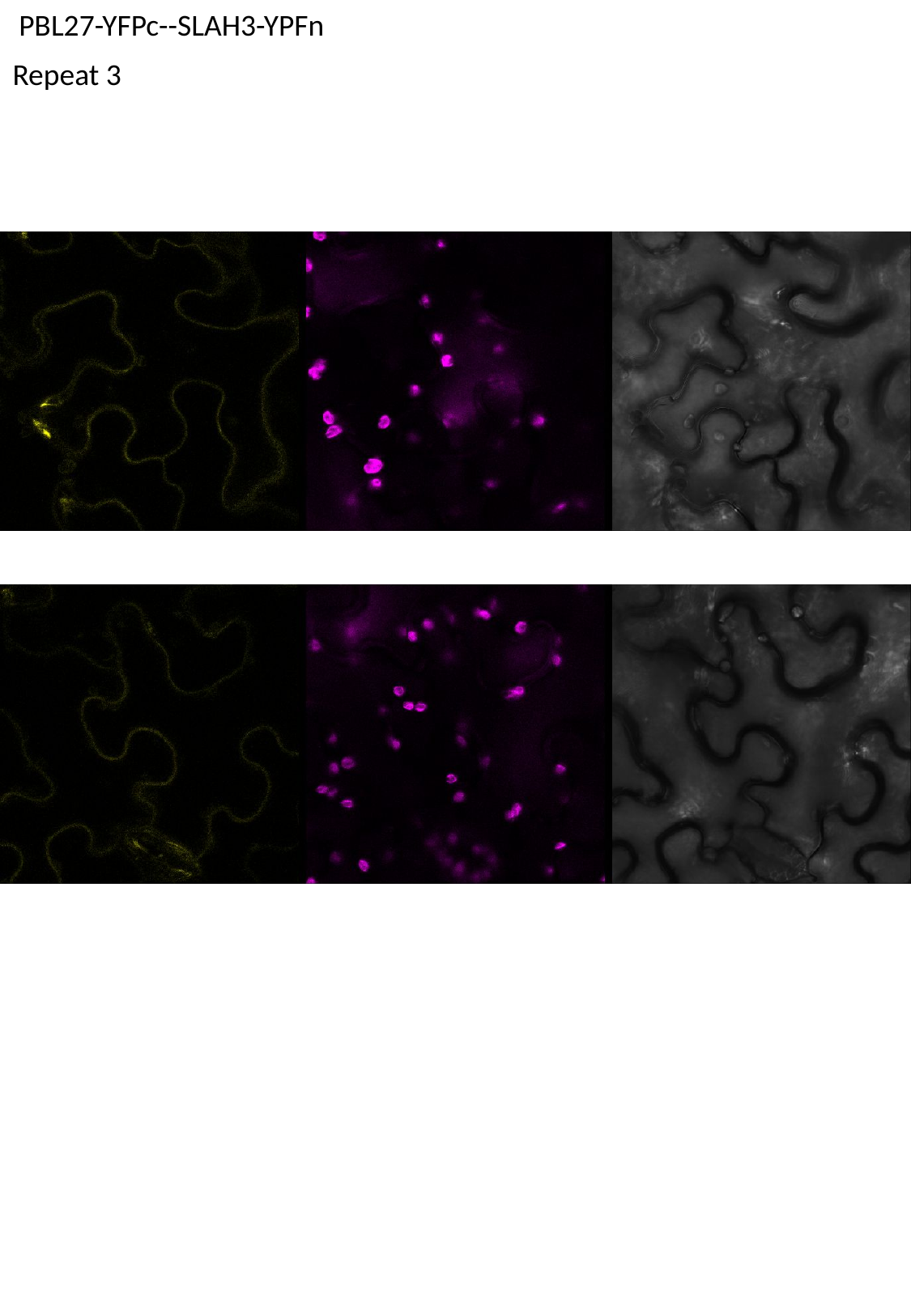

PBL27-YFPc--SLAH3-YPFn
Repeat 3

## Slide 15
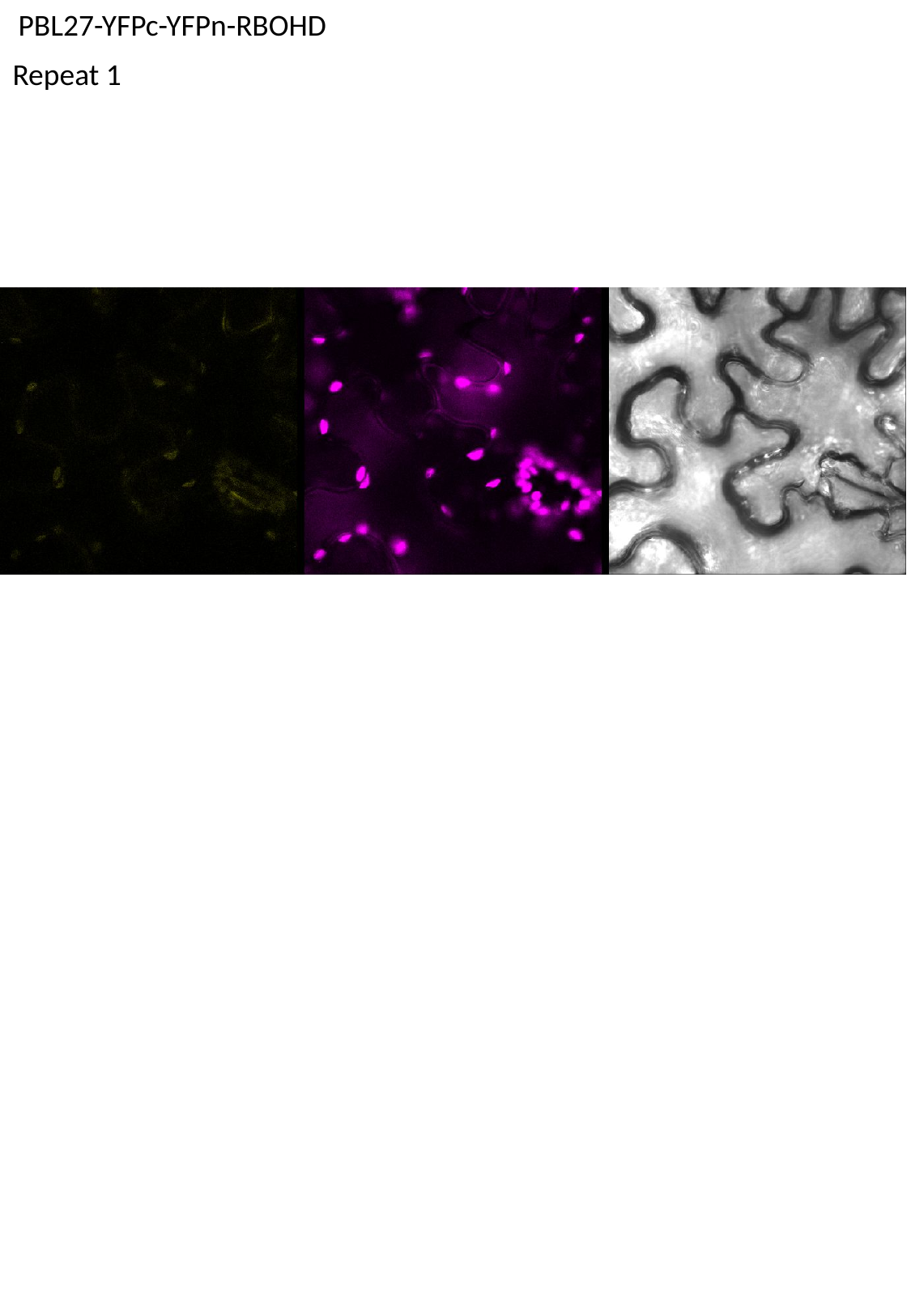

PBL27-YFPc-YFPn-RBOHD
Repeat 1

## Slide 16
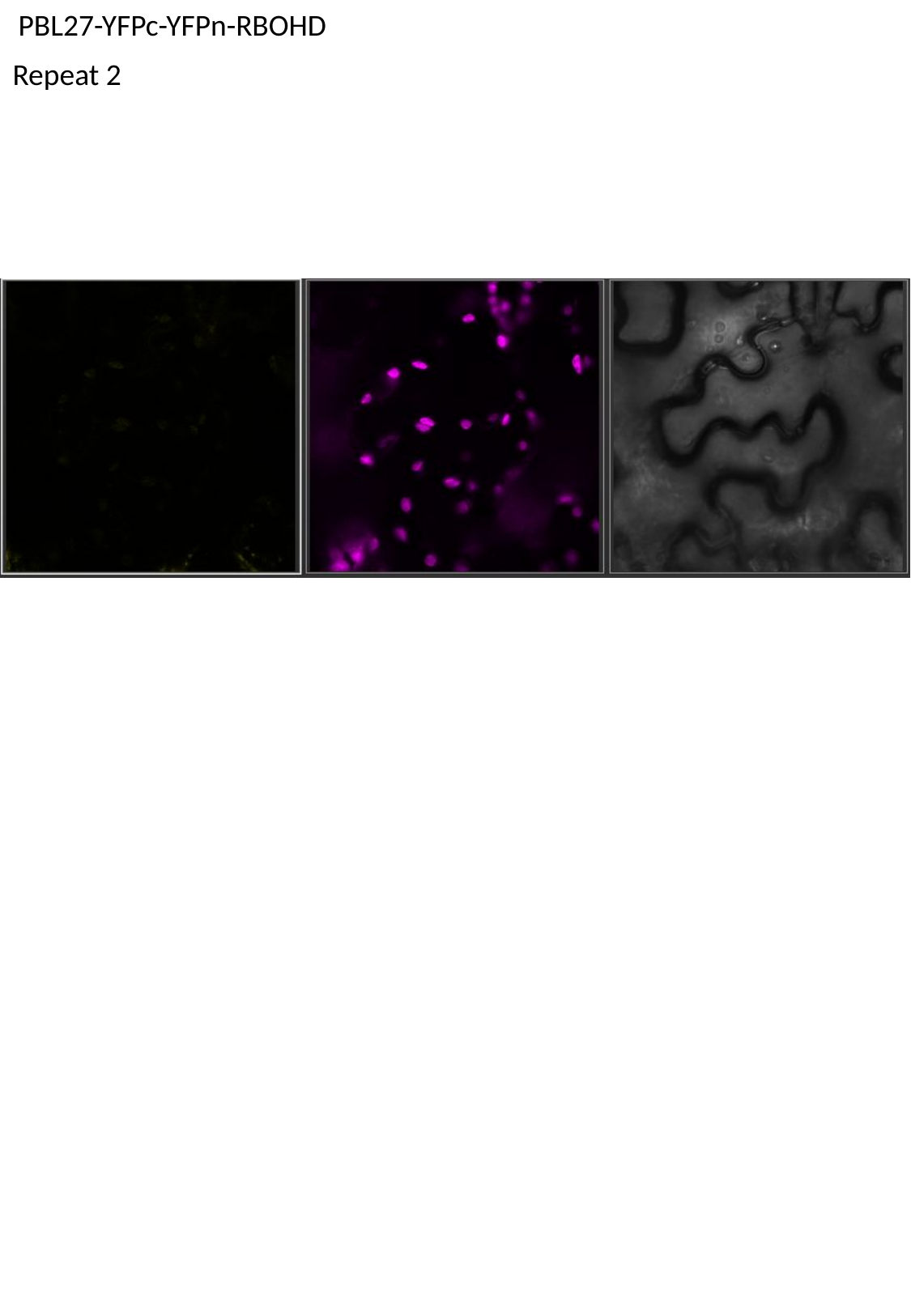

PBL27-YFPc-YFPn-RBOHD
Repeat 2

## Slide 17
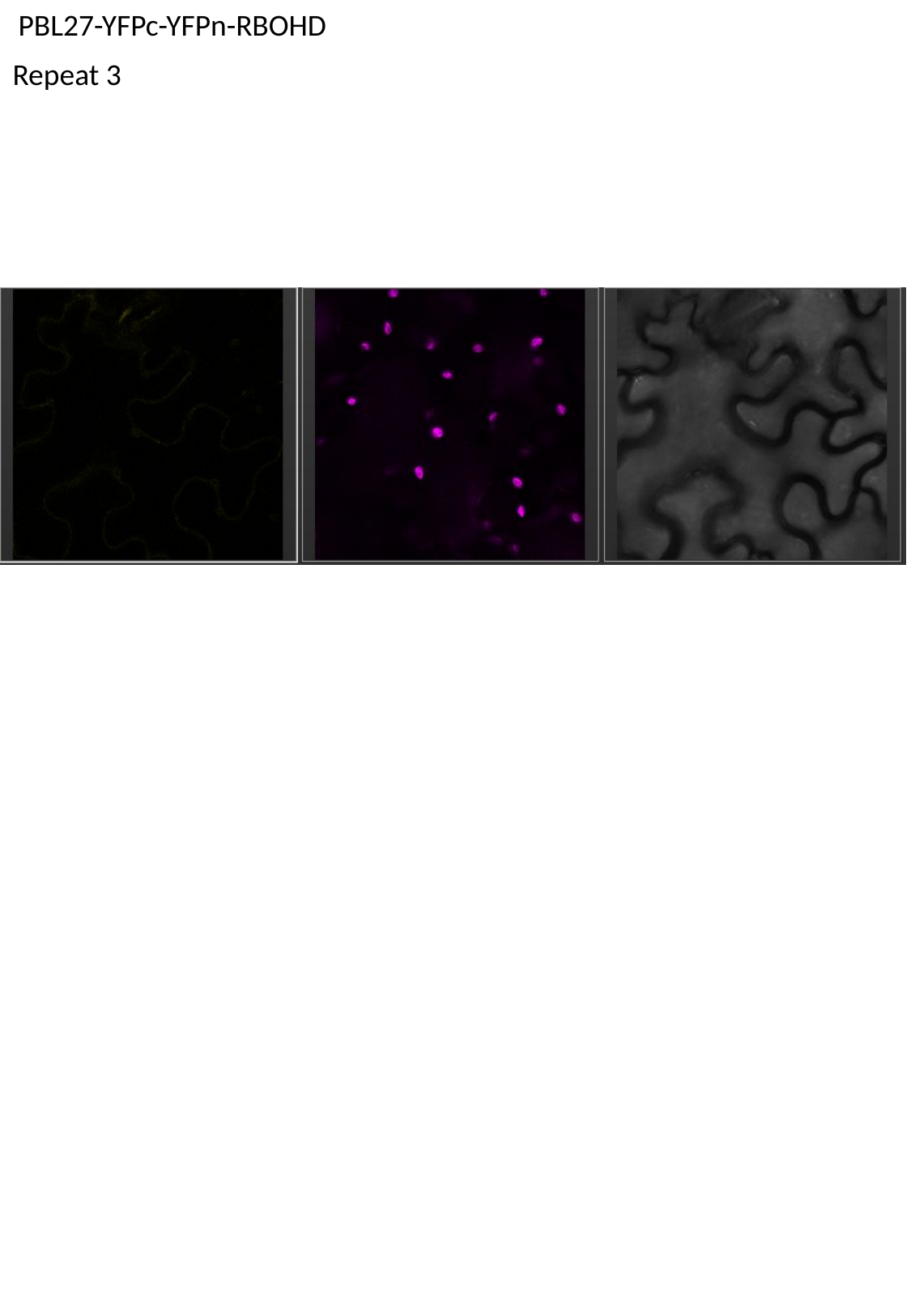

PBL27-YFPc-YFPn-RBOHD
Repeat 3
