# Supplementary material for: Anion channel SLAH3 is a regulatory target of chitin receptor-associated kinase PBL27 in microbial stomatal closure
Source: eLife. 2019 Sep 16;8:e44474. doi: 10.7554/eLife.44474 (PMC6776436; doi:10.7554/eLife.44474)
Supplement: Figure 2—source data 2. [file elife-44474-fig2-data2.pptx]

## Slide 1
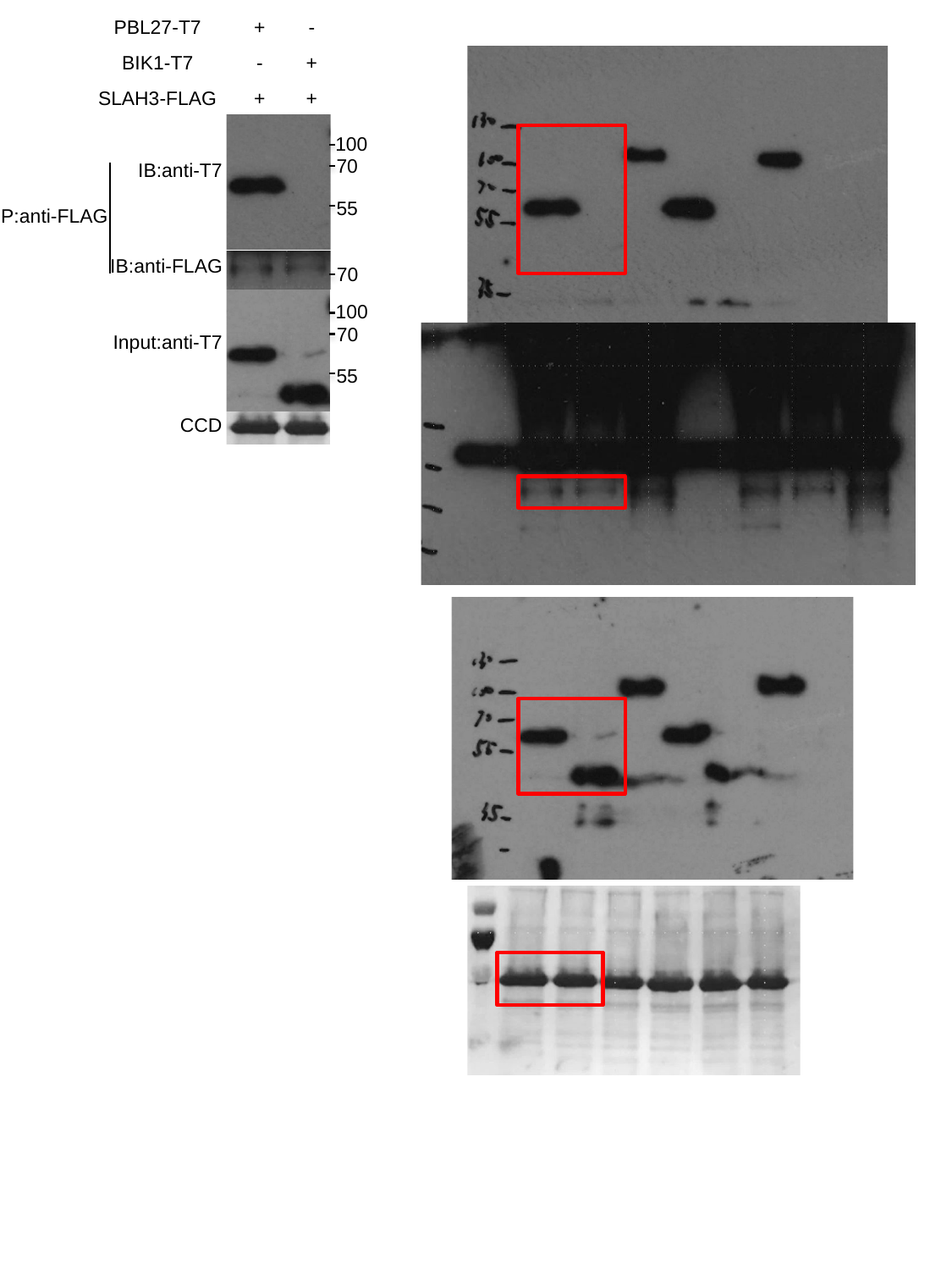

| PBL27-T7 | + | - |
| --- | --- | --- |
| BIK1-T7 | - | + |
| SLAH3-FLAG | + | + |
100
70
IB:anti-T7
55
IP:anti-FLAG
IB:anti-FLAG
70
100
70
Input:anti-T7
55
CCD

## Slide 2
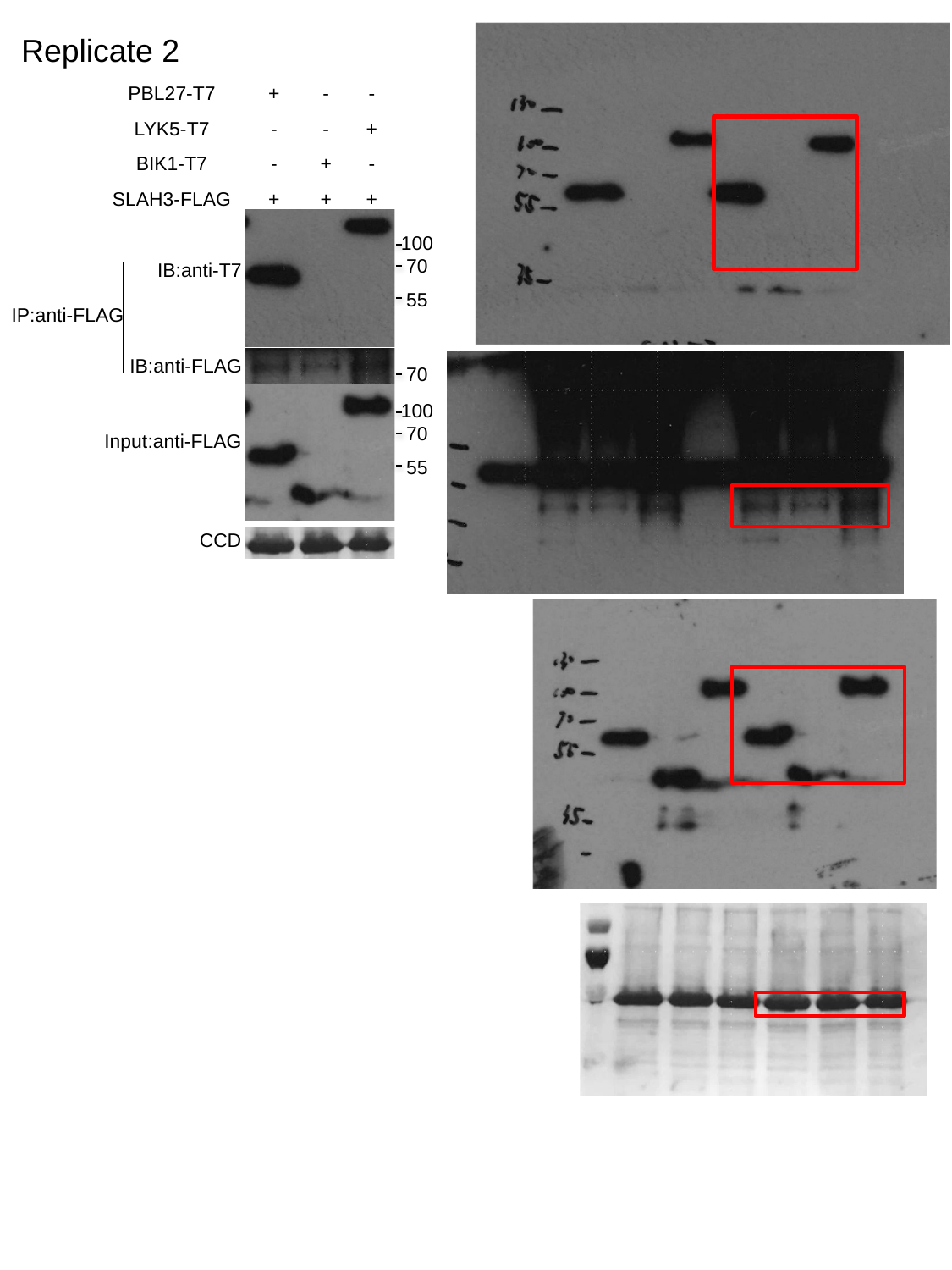

Replicate 2
| PBL27-T7 | + | - | - |
| --- | --- | --- | --- |
| LYK5-T7 | - | - | + |
| BIK1-T7 | - | + | - |
| SLAH3-FLAG | + | + | + |
100
70
IB:anti-T7
55
IP:anti-FLAG
IB:anti-FLAG
70
100
70
Input:anti-FLAG
55
CCD

## Slide 3
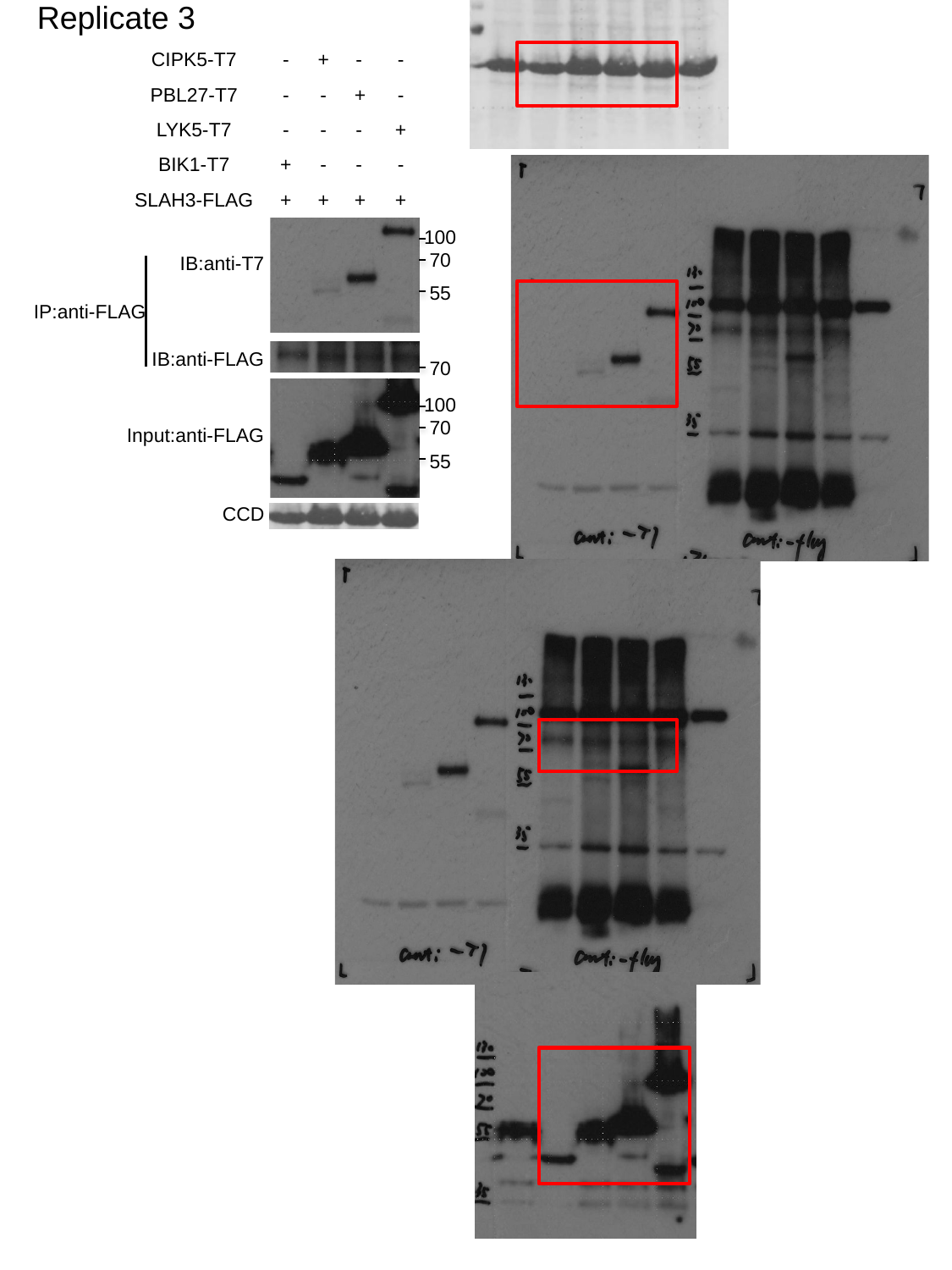

Replicate 3
| CIPK5-T7 | - | + | - | - |
| --- | --- | --- | --- | --- |
| PBL27-T7 | - | - | + | - |
| LYK5-T7 | - | - | - | + |
| BIK1-T7 | + | - | - | - |
| SLAH3-FLAG | + | + | + | + |
100
70
IB:anti-T7
55
IP:anti-FLAG
IB:anti-FLAG
70
100
70
Input:anti-FLAG
55
CCD
