# Supplementary material for: Anion channel SLAH3 is a regulatory target of chitin receptor-associated kinase PBL27 in microbial stomatal closure
Source: eLife. 2019 Sep 16;8:e44474. doi: 10.7554/eLife.44474 (PMC6776436; doi:10.7554/eLife.44474)
Supplement: Figure 2—source data 3. [file elife-44474-fig2-data3.pptx]

## Slide 1
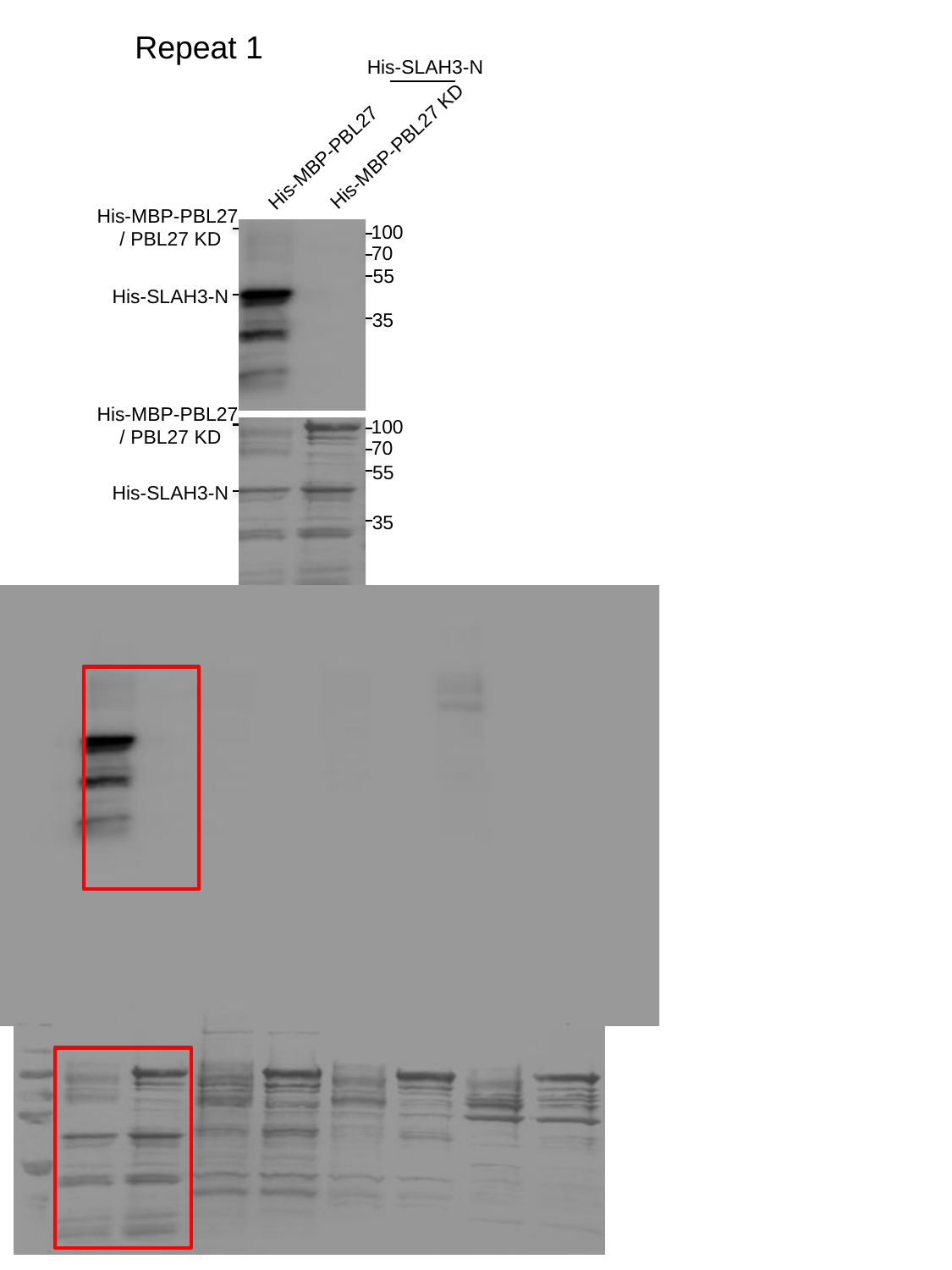

Repeat 1
His-SLAH3-N
His-MBP-PBL27 KD
His-MBP-PBL27
His-MBP-PBL27
/ PBL27 KD
100
70
55
His-SLAH3-N
35
His-MBP-PBL27
/ PBL27 KD
100
70
55
His-SLAH3-N
35

## Slide 2
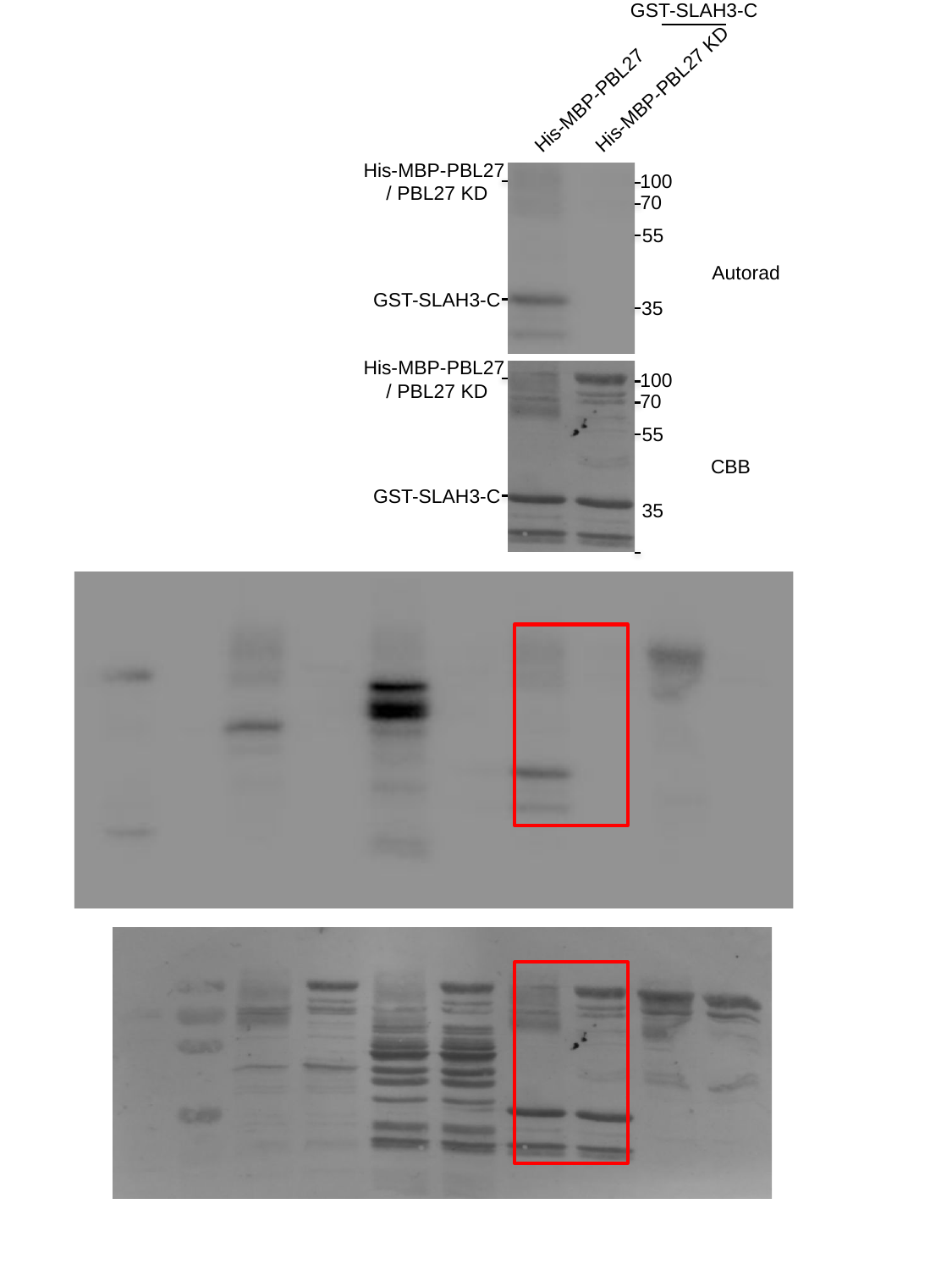

GST-SLAH3-C
His-MBP-PBL27 KD
His-MBP-PBL27
His-MBP-PBL27
/ PBL27 KD
100
70
55
Autorad
GST-SLAH3-C
35
His-MBP-PBL27
/ PBL27 KD
100
70
55
CBB
GST-SLAH3-C
35

## Slide 3
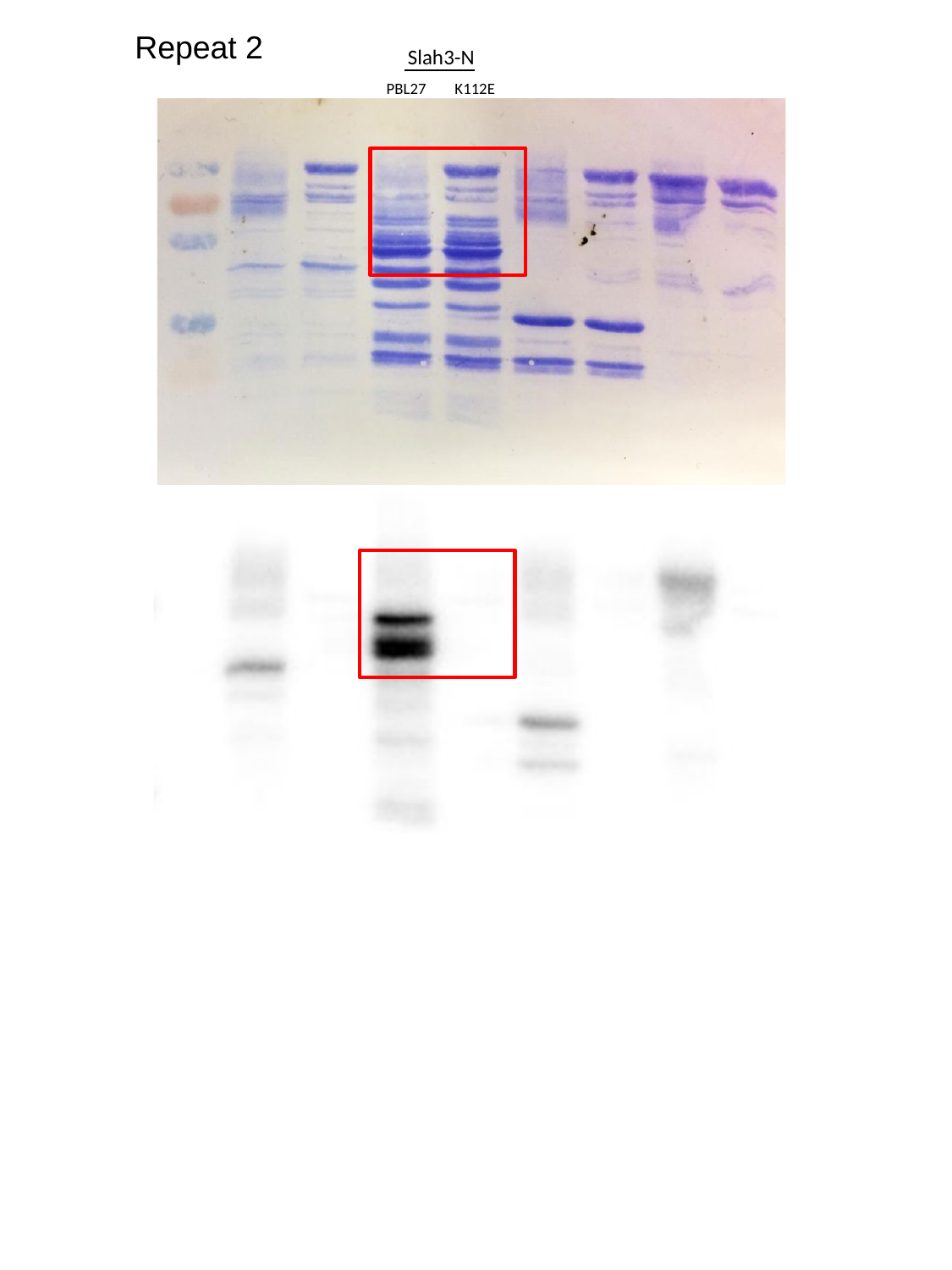

Repeat 2
Slah3-N
 PBL27 K112E

## Slide 4
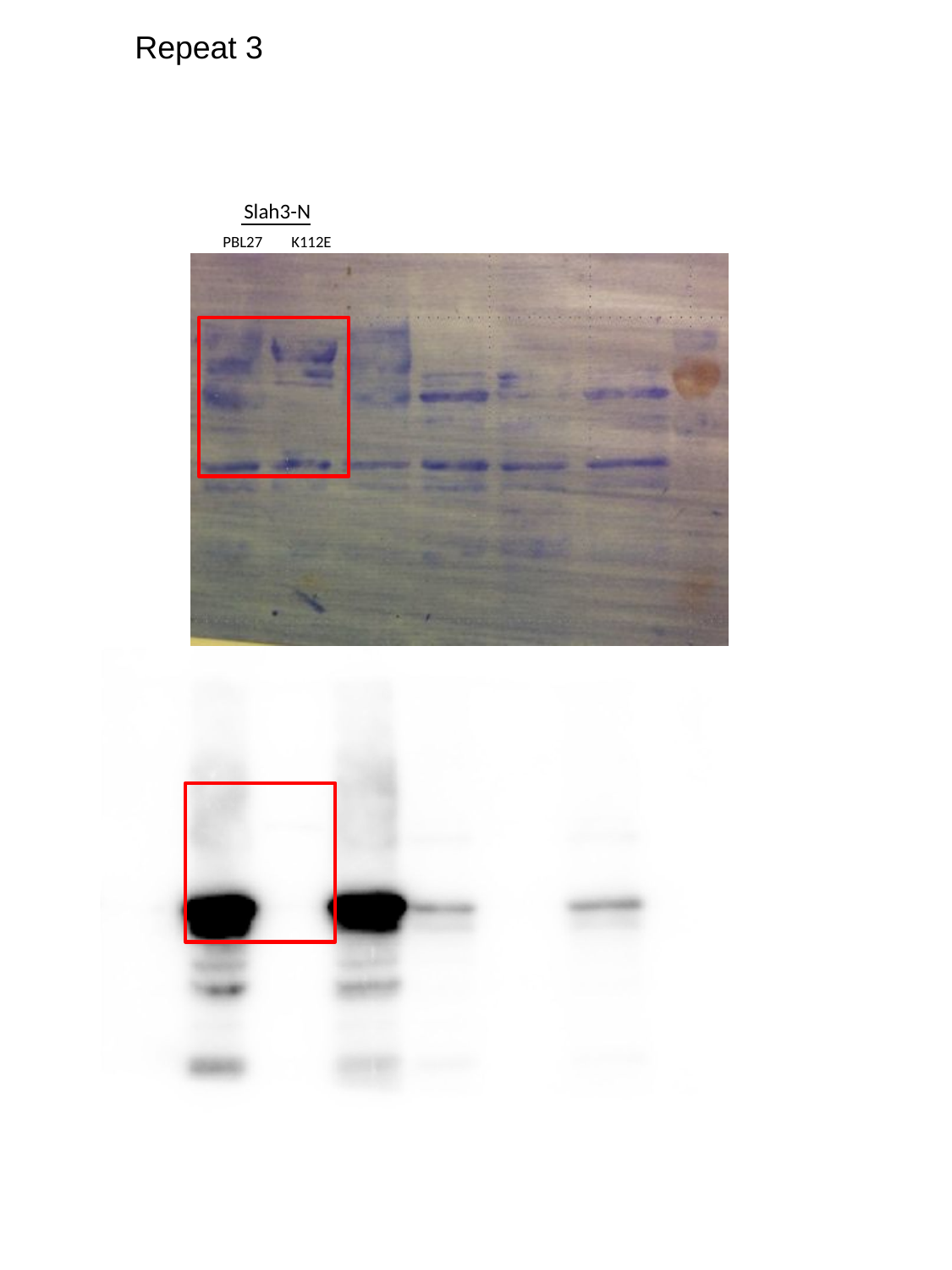

Repeat 3
Slah3-N
 PBL27 K112E
