# Supplementary material for: Anion channel SLAH3 is a regulatory target of chitin receptor-associated kinase PBL27 in microbial stomatal closure
Source: eLife. 2019 Sep 16;8:e44474. doi: 10.7554/eLife.44474 (PMC6776436; doi:10.7554/eLife.44474)
Supplement: Figure 2—source data 4. [file elife-44474-fig2-data4.pptx]

## Slide 1
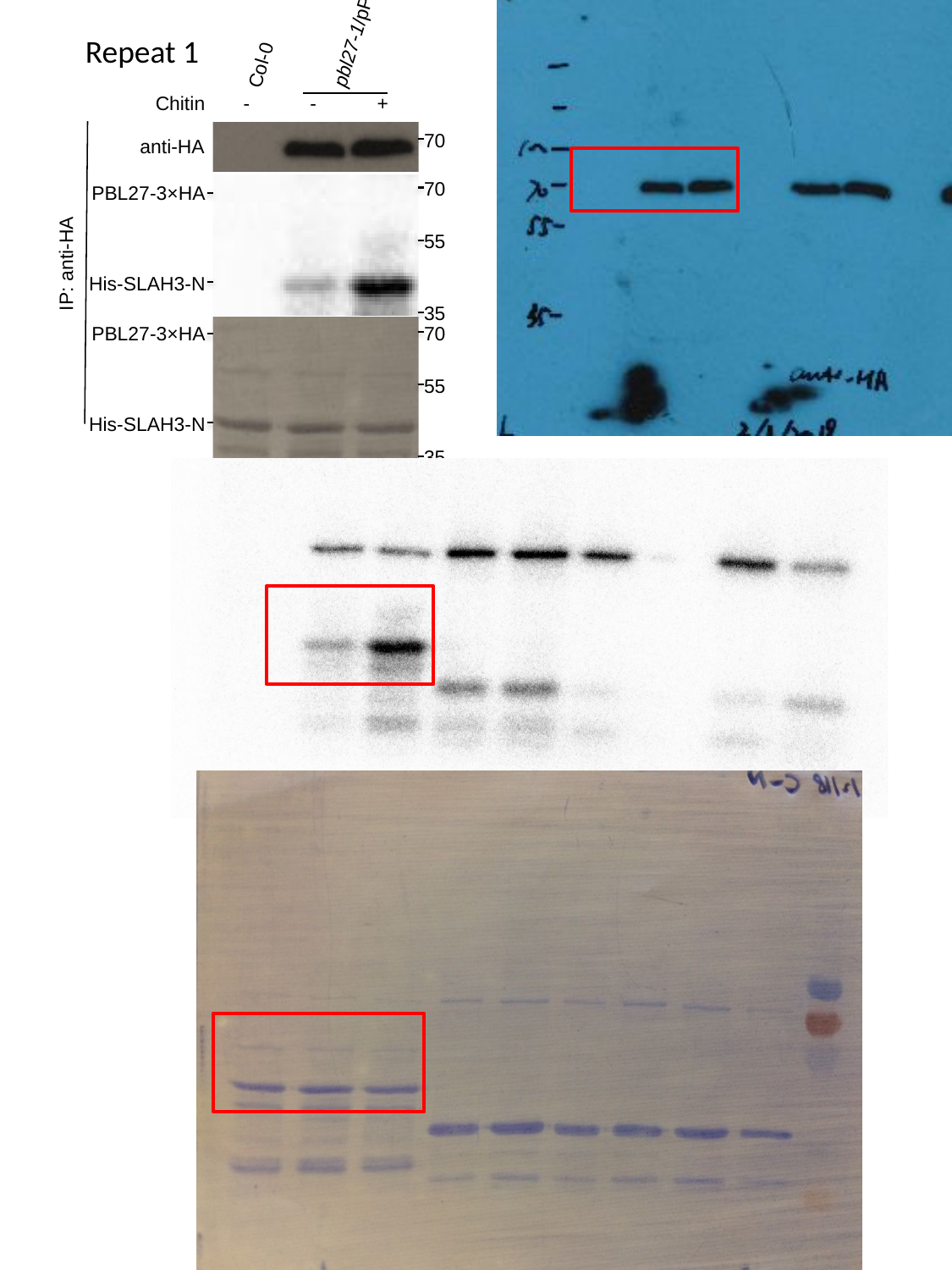

D
pbl27-1/pPBL27:PBL27-3×HA
Repeat 1
Col-0
Chitin - - +
70
anti-HA
70
PBL27-3×HA
IP: anti-HA
55
His-SLAH3-N
35
70
PBL27-3×HA
55
His-SLAH3-N
35

## Slide 2
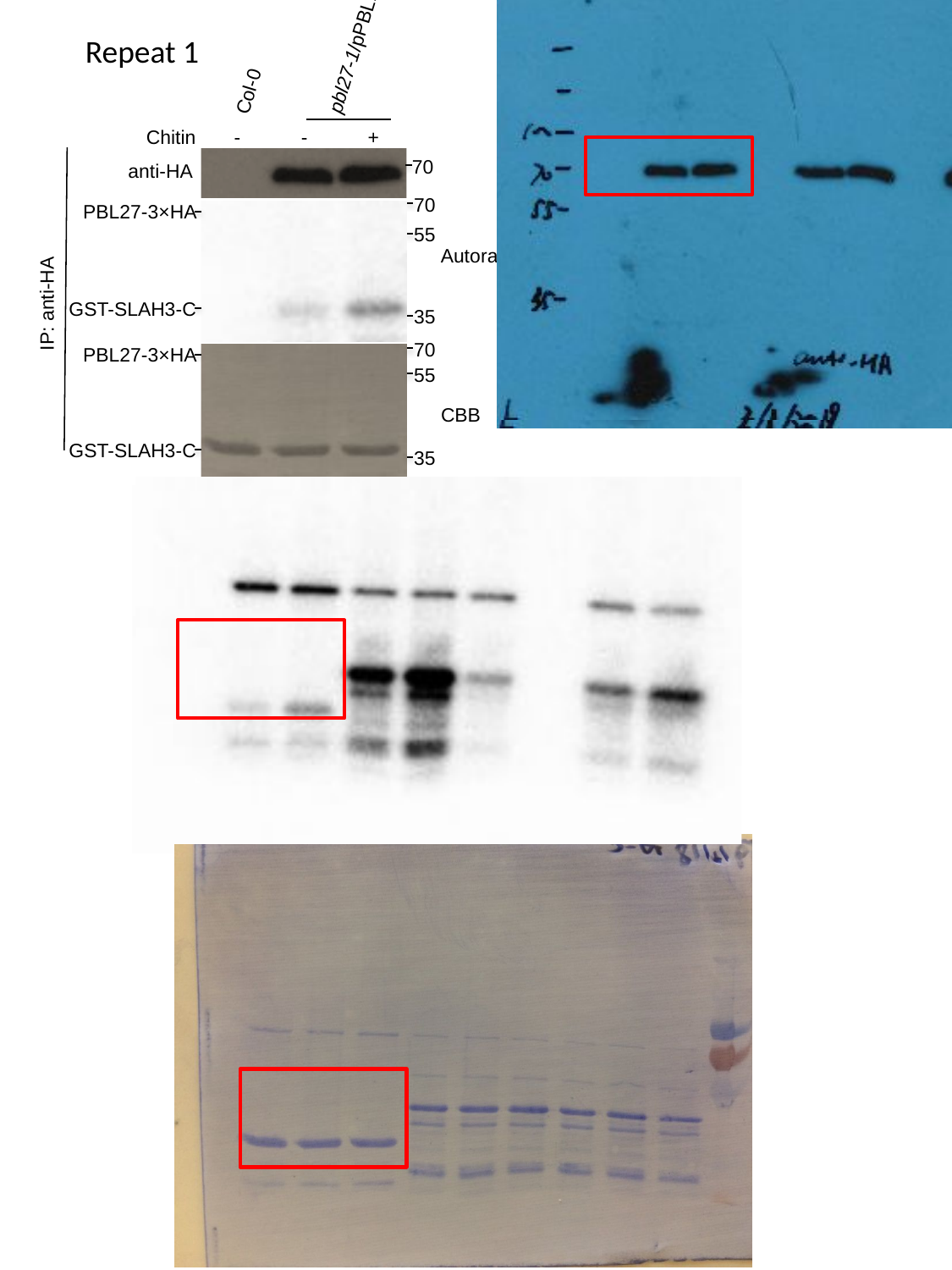

pbl27-1/pPBL27:PBL27-3×HA
Repeat 1
Col-0
Chitin - - +
70
anti-HA
70
PBL27-3×HA
55
Autorad
IP: anti-HA
GST-SLAH3-C
35
70
PBL27-3×HA
55
CBB
GST-SLAH3-C
35

## Slide 3
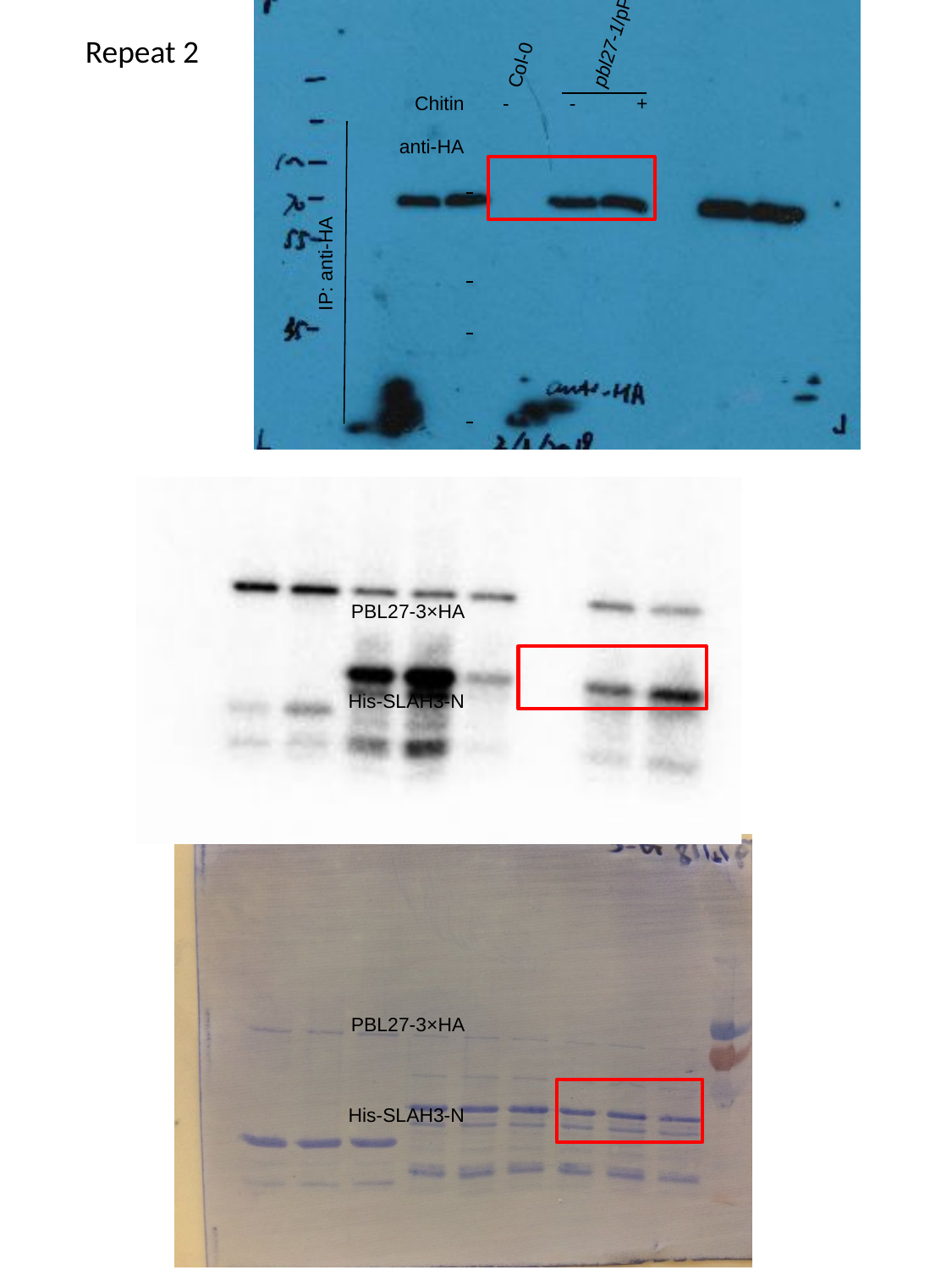

pbl27-1/pPBL27:PBL27-3×HA
Repeat 2
Col-0
Chitin - - +
anti-HA
IP: anti-HA
PBL27-3×HA
His-SLAH3-N
PBL27-3×HA
His-SLAH3-N

## Slide 4
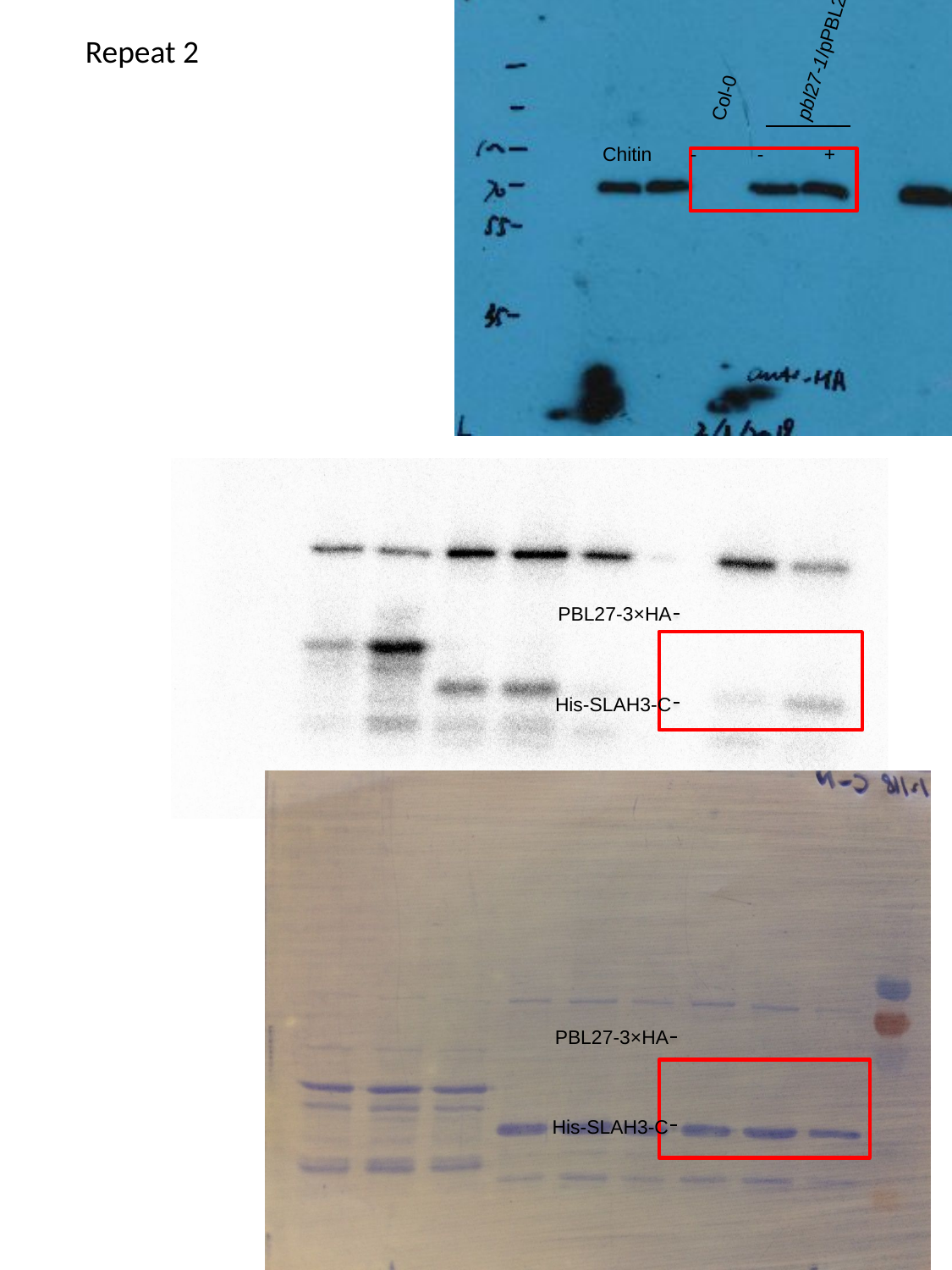

pbl27-1/pPBL27:PBL27-3×HA
Repeat 2
Col-0
Chitin - - +
PBL27-3×HA
His-SLAH3-C
PBL27-3×HA
His-SLAH3-C

## Slide 5
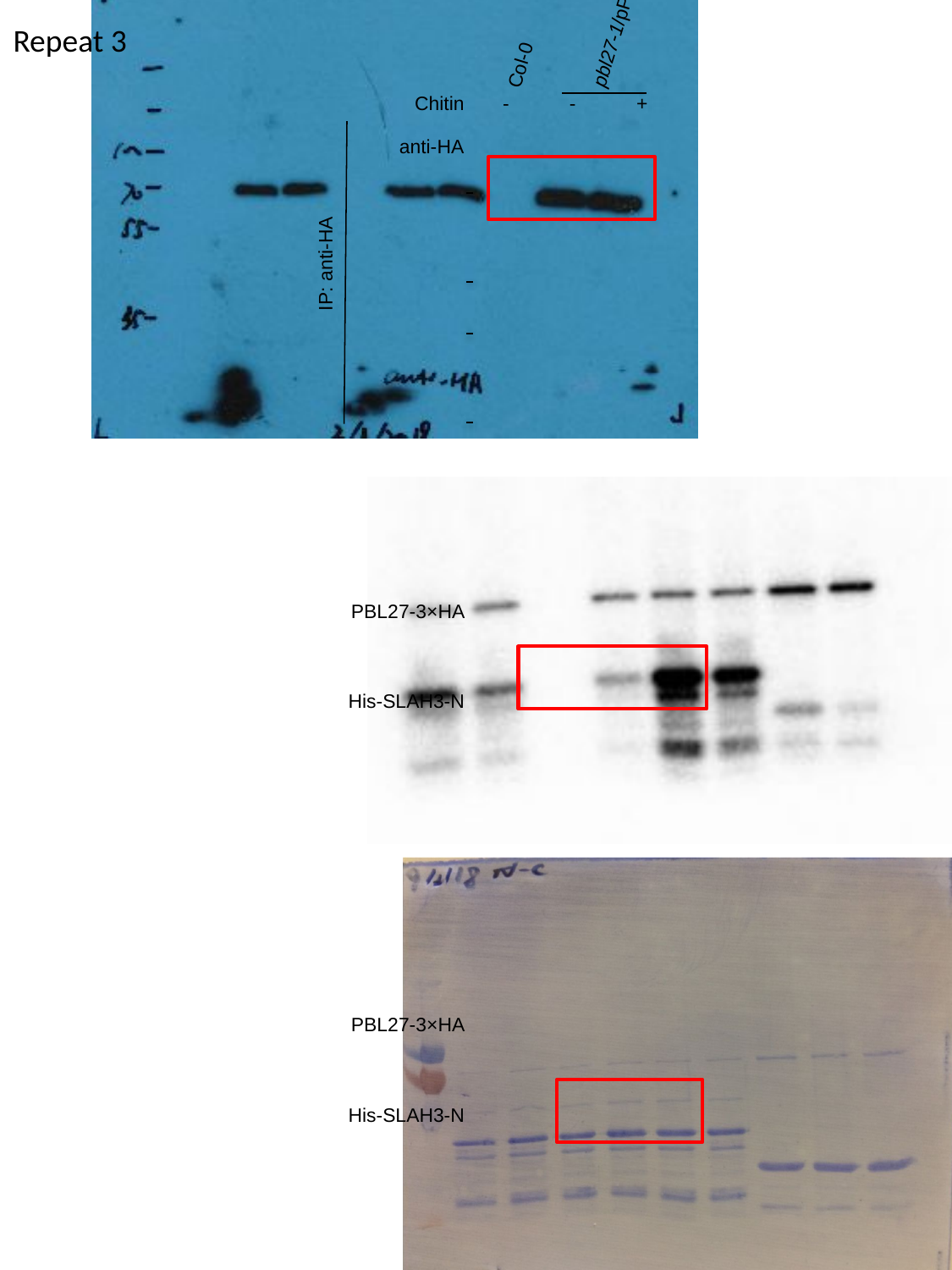

pbl27-1/pPBL27:PBL27-3×HA
Repeat 3
Col-0
Chitin - - +
anti-HA
IP: anti-HA
PBL27-3×HA
His-SLAH3-N
PBL27-3×HA
His-SLAH3-N

## Slide 6
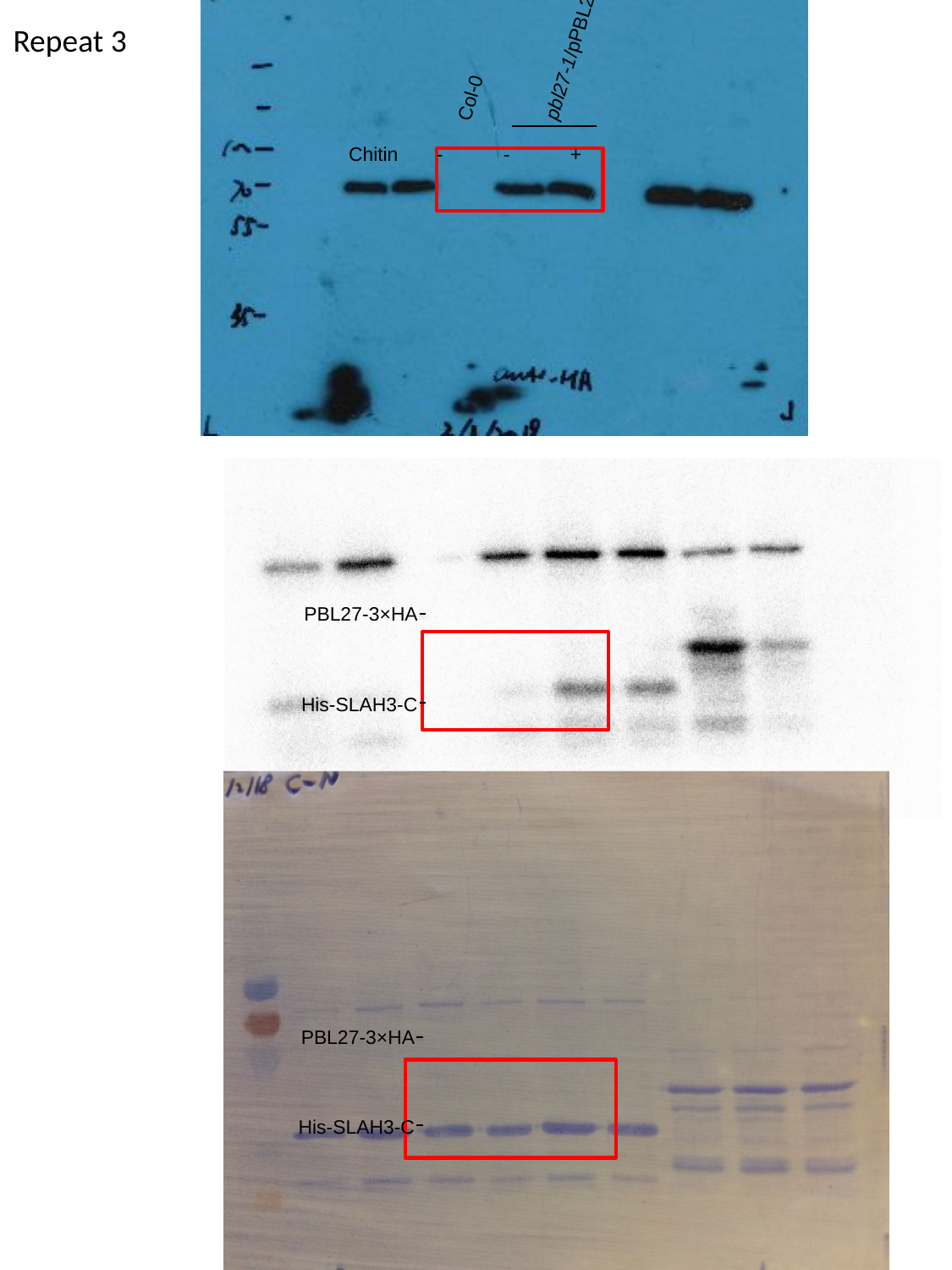

pbl27-1/pPBL27:PBL27-3×HA
Repeat 3
Col-0
Chitin - - +
PBL27-3×HA
His-SLAH3-C
PBL27-3×HA
His-SLAH3-C
