# Supplementary material for: Anion channel SLAH3 is a regulatory target of chitin receptor-associated kinase PBL27 in microbial stomatal closure
Source: eLife. 2019 Sep 16;8:e44474. doi: 10.7554/eLife.44474 (PMC6776436; doi:10.7554/eLife.44474)
Supplement: Figure 2—source data 5. [file elife-44474-fig2-data5.pptx]

## Slide 1
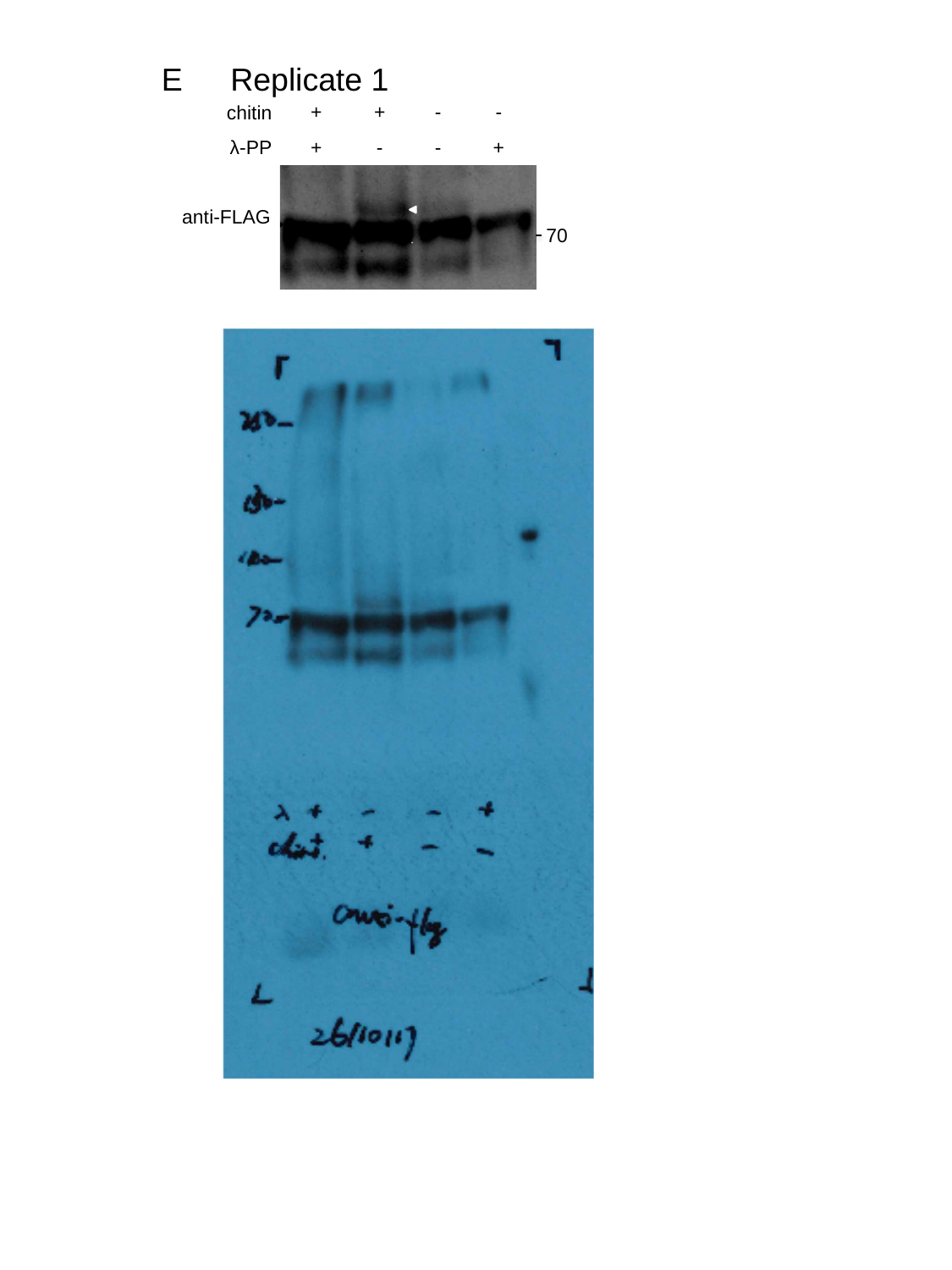

Replicate 1
E
| chitin | + | + | - | - |
| --- | --- | --- | --- | --- |
| λ-PP | + | - | - | + |
anti-FLAG
70

## Slide 2
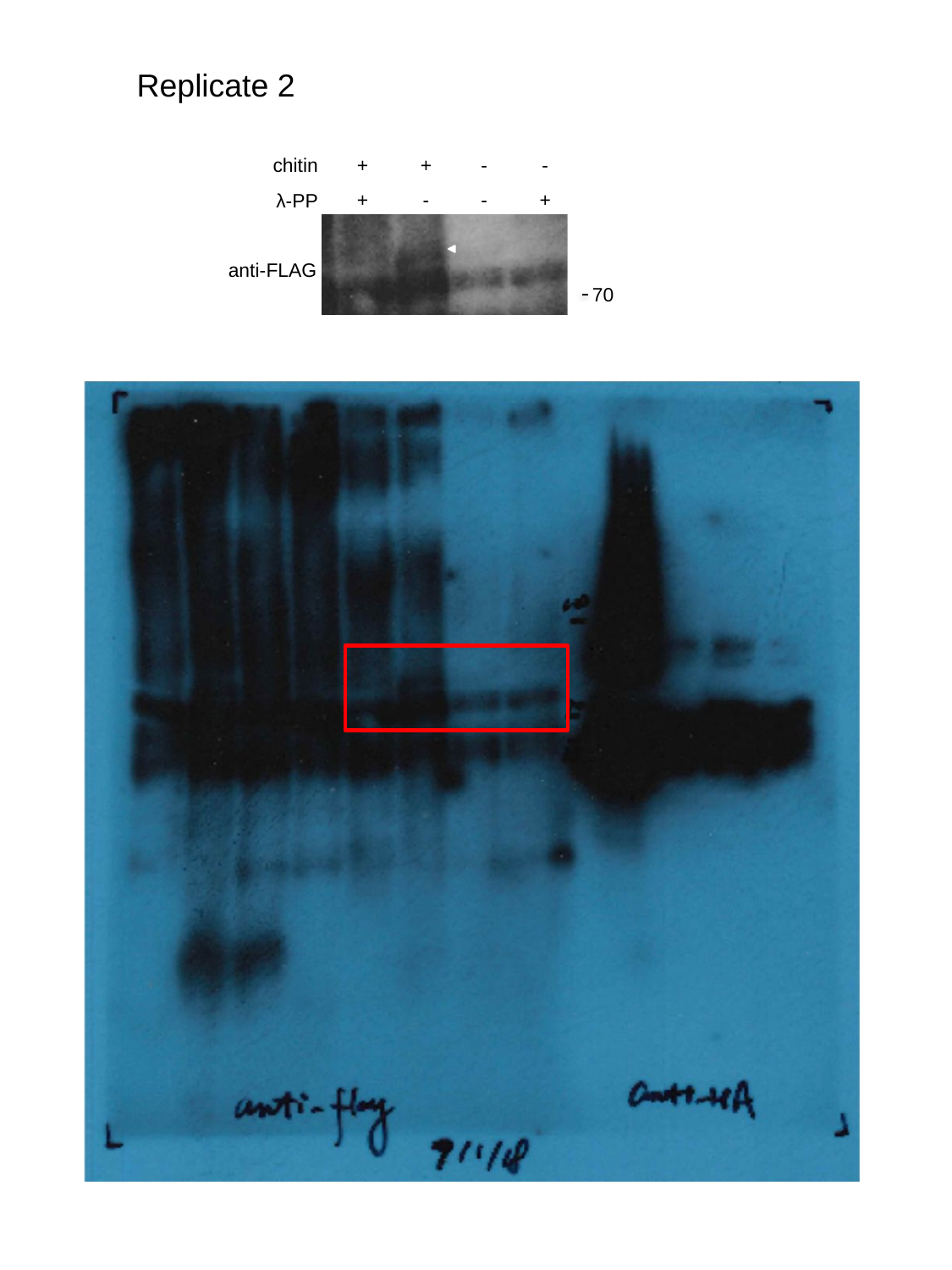

Replicate 2
| chitin | + | + | - | - |
| --- | --- | --- | --- | --- |
| λ-PP | + | - | - | + |
anti-FLAG
70

## Slide 3
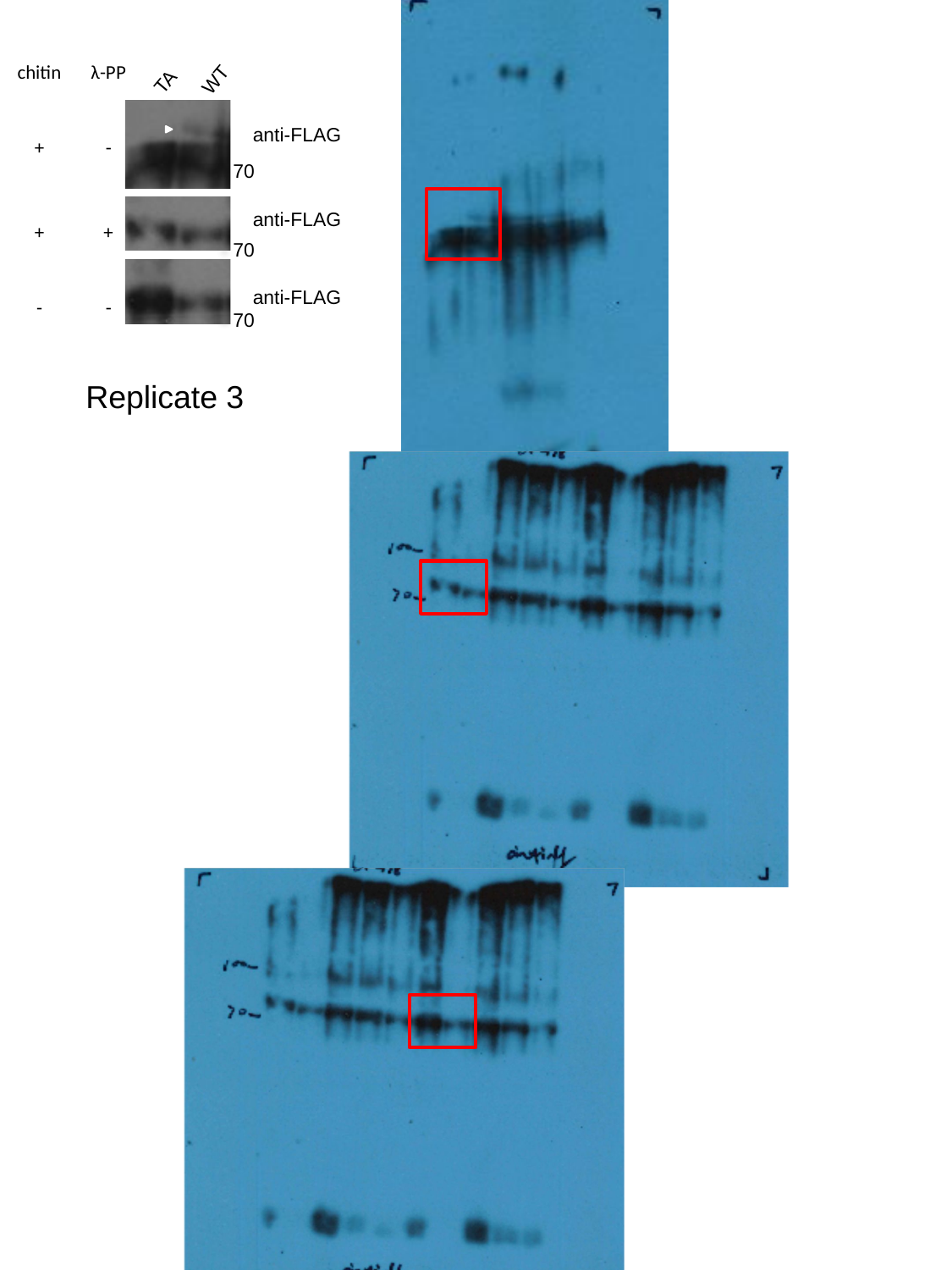

| chitin | λ-PP |
| --- | --- |
| + | - |
| + | + |
| - | - |
WT
TA
anti-FLAG
70
anti-FLAG
70
anti-FLAG
70
Replicate 3
